# Phosphorylation-Independent Regulation of the Diguanylate Cyclase WspR

**DOI:** 10.1371/journal.pbio.0060067

**Published:** 2008-03-25

**Authors:** Nabanita De, Michelle Pirruccello, Petya Violinova Krasteva, Narae Bae, Rahul Veera Raghavan, Holger Sondermann

**Affiliations:** 1 Department of Molecular Medicine, College of Veterinary Medicine, Cornell University, Ithaca, New York, United States of America; 2 Department of Molecular and Cell Biology, University of California, Berkeley, Berkeley, California, United States of America; Harvard Medical School, United States of America

## Abstract

Environmental signals that trigger bacterial pathogenesis and biofilm formation are mediated by changes in the level of cyclic dimeric guanosine monophosphate (c-di-GMP), a unique eubacterial second messenger. Tight regulation of cellular c-di-GMP concentration is governed by diguanylate cyclases and phosphodiesterases, which are responsible for its production and degradation, respectively. Here, we present the crystal structure of the diguanylate cyclase WspR, a conserved GGDEF domain-containing response regulator in Gram-negative bacteria, bound to c-di-GMP at an inhibitory site. Biochemical analyses revealed that feedback regulation involves the formation of at least three distinct oligomeric states. By switching from an active to a product-inhibited dimer via a tetrameric assembly, WspR utilizes a novel mechanism for modulation of its activity through oligomerization. Moreover, our data suggest that these enzymes can be activated by phosphodiesterases. Thus, in addition to the canonical pathways via phosphorylation of the regulatory domains, both product and enzyme concentration contribute to the coordination of c-di-GMP signaling. A structural comparison reveals resemblance of the oligomeric states to assemblies of GAF domains, widely used regulatory domains in signaling molecules conserved from archaea to mammals, suggesting a similar mechanism of regulation.

## Author Summary

Bacteria can switch from a single-cell, free-floating behavioral mode to a community life-form via colonization of surfaces and the secretion of an extracellular matrix. This process, called biofilm formation, has been attributed to a majority of chronic infections, including the lungs, as occurs in patients with cystic fibrosis. Recently, a small intracellular signaling molecule, the nucleotide cyclic dimeric guanosine monophosphate (c-di-GMP), and enzymes for its production and degradation have been discovered that relay environmental cues to changes in secretion, cell adhesion and ultimately, biofilm formation and virulence. We have studied the molecular mechanism and mode of regulation of WspR, an enzyme from *Pseudomonas* and related pathogenic bacteria responsible for the generation of c-di-GMP and biofilm formation. On the basis of its crystal structure and functional assays, we elucidated a sophisticated regulatory mechanism in WspR that is controlled by feedback inhibition mediated by c-di-GMP. We hypothesize that WspR is primed for the (re)activation by enzymatic degradation of the inhibitory nucleotide. In addition, we identified mutations at the inhibitory site of WspR in a subset of bacteria that are frequently found in cystic fibrosis patients, suggesting that altered c-di-GMP signaling, mediated by modified WspR, may contribute to the pathogenicity of these strains. Furthermore, we present a structural comparison with GAF domains, which are widely used conserved regulatory signaling domains, suggesting a similar mechanism of regulation.

## Abstract 3

We present a model for the regulation of a conserved diguanyate cyclase from*Pseudomonas* that is responsible for cyclic di-GMP production and biofilm formation, providing insight into the molecular mechani7sm controlling cell signaling and virulence.

## Introduction

Bacterial signal transduction pathways regulating virulence and chemotaxis are commonly composed of two-component signaling systems. These systems consist of a histidine protein kinase that relays environmental stimuli to phosphotransfer reactions, ultimately controlling the activity of phospho-receiver domain-containing response regulator proteins [1]. These phosphorylation-activated switches have been described as undergoing rather simple, reversible changes in oligomerization state and/or conformation, and are often presented as modular proteins with a regulatory phospho-receiver domain being linked to an effector domain [2].

An emerging family of response regulators controls the cellular level of the bacterially unique second messenger, bis-(3′-5′)-cyclic dimeric guanosine monophosphate (cyclic di-GMP or c-di-GMP) [3–6]. Enzymes responsible for c-di-GMP synthesis and degradation have been identified in most eubacteria, being absent in archaea and eukaryotes [7]. GGDEF and EAL describe the consensus sequence motif in the active site of these enzymes. Diguanylate cyclases of the GGDEF family catalyze the cyclization of two guanosine triphosphate (GTP) molecules to one c-di-GMP molecule [8,9], and have been shown to be homologous to adenylate cyclases [10]. Phosphodiesterase (PDE) activity responsible for the breakdown of c-di-GMP has been demonstrated for EAL domain-containing proteins of an unknown fold [11–13].

Levels of c-di-GMP trigger cellular responses relevant to pathogenesis, such as in motility, secretion, cytotoxicity, and biofilm formation, a differentiation process by which bacteria switch from a planktonic, single-cell–based suspension to a sessile community life-form [14]. Biofilms have been associated with chronic infections, for example of the ear, heart, or lungs, especially in patients suffering from cystic fibrosis. Biofilms often induce tolerance or resistance to host defense and antibiotic treatment [15]. Enhanced biofilm formation has been attributed to high cellular c-di-GMP concentration, whereas low levels of c-di-GMP can lead to an impairment of biofilm formation and cytotoxicity [16–18], suggesting that cellular c-di-GMP levels, and thus the activity of c-di-GMP–specific cyclases and PDEs, are under tight control.

Mechanistic information regarding the modes of regulation of GGDEF and EAL domain-containing proteins is sparse. Structural models are only available for the diguanylate cyclase PleD from Caulobacter crescentus [19], consisting of two CheY-homology phospho-receiver domains and a GGDEF domain (Figure S3B and S3C). In the structure of unphosphorylated PleD, the first CheY-homology domain containing the phosphorylation switch is in an inactive conformation [19]. Cyclic di-GMP is bound to the catalytic site as well as to a second site distal to the catalytic loop. The latter is composed of a motif on the GGDEF domain (RxxD motif) and the second CheY-homology domain. Subsequently, the RxxD motif has been shown to be a conserved allosteric inhibitory site (I-site) in GGDEF domain-containing proteins [20]. A model for regulation by phosphorylation and dimerization has been proposed for PleD with c-di-GMP serving as a noncompetitive inhibitor [21]. A recent crystal structure of PleD bound to the phosphoryl analog beryllium fluoride (BeF_3_^−^) confirms such a mechanism [22]. The structure also highlights a second mode of product inhibition in which c-di-GMP bridges two cyclase domains in the activated dimer.

**Figure 1:**
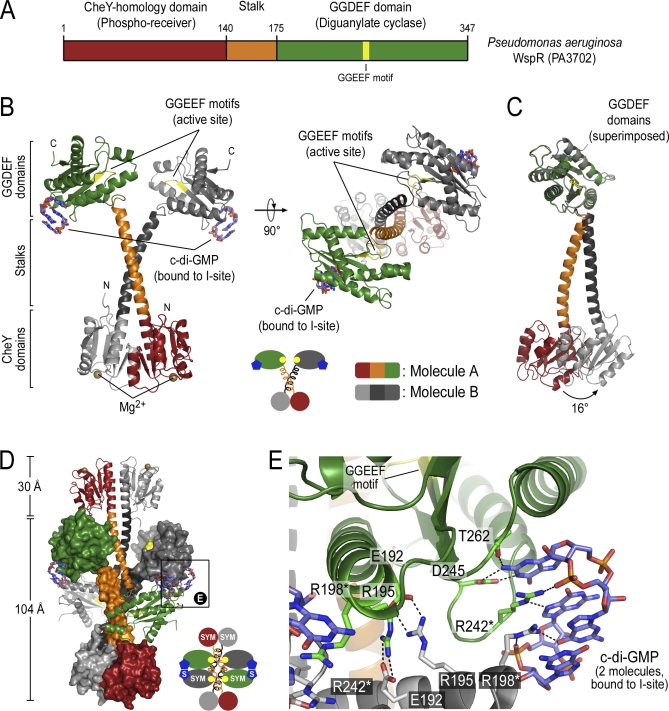
Structure of WspR from P. aeruginosa (A) Domain organization of WspR. The N-terminal CheY-homology phospho-receiver domain is connected via a helical stalk to the GGDEF domain with diguanylate cyclase activity. In WspR, the active site loop contains the GGEEF motif (residues 251–255). (B) The crystal structure of WspR. The crystals contain two molecules in the asymmetric unit. Two orthogonal views are shown with coloring for molecule A as shown in (A). Molecule B is colored grey. The GGEEF motif is shown in yellow. Cyclic di-GMP molecules bound to the inhibitory site (I-site) are located distal to the active site and are shown as sticks. Mg^2+^ ions are shown as brown spheres. (C) Comparison of the two WspR molecules in the asymmetric unit. Molecules A and B were aligned through superpositioning of their GGDEF domains. The CheY-stalk modules are separated by a rigid body rotation of 16° around residue 172 at the tip of the helical stalk. (D) Crystallographic tetramer consisting of two symmetry-related dimers representing a biological unit. Two C2-symmetry–related crystallographic dimers of WspR are shown intertwined in a head-to-head orientation. The stalks form a tetrameric structure splaying apart the coiled-coils and physically blocking the active sites. Cyclic di-GMP molecules bound at the I-site bridge the GGDEF domains of neighboring molecules. “S” or “SYM” in the cartoon diagram indicate crystal symmetry-related molecules. The boxed region is shown in (E). (E) Close-up view of the I-site. In the crystal, two intercalated c-di-GMP molecules are bound at the I-site located at the back of the GGDEF domain distal to the catalytic site. An arginine side chain (R198) contributed by a symmetry-related GGDEF domain completes the I-site. Asterisks indicate residues targeted for site-directed mutagenesis.

WspR (PA3702 in Pseudomonas aeruginosa), often described as an ortholog of PleD, is a conserved response regulator of a chemosensory signaling system controlling biofilm formation and other adaptive phenotypic changes in *Pseudomonas* and related species [23,24] (Figure S1). Overexpression of WspR causes hyperbiofilm formation, whereas loss-of-function mutants show reduced biofilm formation and cytotoxicity [17], suggesting that WspR is a potent switch controlling virulence mechanisms. WspR has a similar domain organization as PleD; however, it lacks the second CheY-homology domain (Figure 1A). As observed with PleD, WspR appears to be regulated by phosphorylation of the N-terminal CheY-homology phospho-receiver domain [24]. A recent systematic mutagenesis study identified other functionally important regions in WspR, but the exact mechanism of regulation remains unknown [25].

Here, we present the crystal structure of WspR from P. aeruginosa with c-di-GMP bound at its inhibitory site. WspR is trapped in the crystal in a distinct inactive state that suggests a novel mechanism of feedback inhibition and activation. Crystal symmetry–related molecules form a catalytically inactive tetrameric assembly mediated predominantly by an antiparallel packing of helical stalks, coiled-coil motifs connecting the regulatory CheY modules to the GGDEF domains. Unexpectedly, in solution, the product-inhibited conformation corresponds to an elongated dimer. Upon c-di-GMP hydrolysis by PDE treatment, the conformation switches to a more compact dimer with high activity. We show that the tetrameric species serves as a scaffold for the formation of the inhibited, elongated state from the active, more compact state, and is a required intermediate for establishing c-di-GMP–mediated inhibition. Hence, the tetrameric assembly observed in the crystal covers features of both dimeric states. Although some of the structural hallmarks are similar to the ones observed for PleD [19,22], we propose a novel, phosphorylation-independent mode of regulation for WspR, based on a biochemical characterization of the inhibition mechanism.

## Results/Discussion

### Crystal Structure of WspR from P. aeruginosa

We determined the crystal structure of full-length WspR from P. aeruginosa containing a CheY-homology and GGDEF domain at 2.4 Å resolution (Figure 1; Table S1). The structure was solved by molecular replacement using the isolated CheY (D1) and GGDEF domains of C. crescentus PleD as search models [19]. Electron density maps, phased by molecular replacement, are of good quality and reveal novel features that were built with confidence (Figure S2 and Table S1). The asymmetric unit contains two molecules with similar conformation that only differ by a 16° rigid body rotation with respect to the relative orientation of the GGDEF domain and the N-terminal regulatory unit (Figure 1B and 1C).

In the crystal lattice, WspR forms a tetramer by an antiparallel packing of two WspR dimers (Figure 1B and 1D; “S” or “SYM” in the diagrams indicates crystal symmetry–related molecules). In these dimers representing the asymmetric unit, the CheY-homology domains adopt a conformation reminiscent of an active phospho-receiver dimer observed in OmpR/PhoB-type response regulators [26] (Figure S3A). Due to the lack of posttranslational modifications (see Material and Methods), and considering the coordination geometry, extra density near the conserved aspartates at the active site of the CheY domains was interpreted as Mg^2+^ ions (Figure 1B). The C-terminal helices of the CheY-homology domains, referred to as stalks, were clearly resolved in the electron density maps after molecular replacement, and extend to long coiled-coil–like structures that connect to the GGDEF domains (Figure S2A). The GGDEF domains are oriented such that the two active sites face each other, similar to conformations of active adenylate cyclases [27] (Figure 1B). In addition, the electron density maps clearly revealed binding of an intercalated c-di-GMP dimer to a conserved inhibitory site (I-site) [20] (Figure S2B). The conformation of the nucleotides and their mode of interaction with the GGDEF domain are similar both to small-molecule crystal structures of c-di-GMP and the conformation seen when bound to PleD [19,22,28,29] (Figure 1B and 1E).

Although the structure of WspR represents a similar functional state as the c-di-GMP–bound conformations of PleD and highlights conservation of regulatory features [19,20], it reveals major structural differences that suggest a distinct mechanism of product inhibition (Figure S3B and S3C). In monomeric PleD, c-di-GMP binds both to the I-site located at the GGDEF domain and the second CheY-homology domain (D2) [19]. Whereas the two CheY-homology domains of PleD (D1-D2; including their protruding terminal helices) superimpose well with the phospho-receiver dimer of WspR, their position relative to the GGDEF domains is markedly different (Figure S3B and S3C). In addition, WspR lacks the second phospho-receiver domain that is part of the I-site in PleD, suggesting that c-di-GMP might mediate product inhibition of WspR by a different mechanism. In the dimeric state of PleD, activated by BeF_3_^−^, product inhibition is achieved by I-site–bound c-di-GMP bridging the GGDEF domains, locking them in an inactive conformation [22] (Figure S3C). This feature is conserved in the WspR structure but is part of a vastly different regulatory mechanism.

Cyclic di-GMP at the I-site is bound to two conserved arginine residues, R242 and R198, the latter being contributed by a symmetry-related GGDEF domain (Figure 1E). The GGDEF dimer conformation and mode of c-di-GMP binding is similar to that of activated, dimeric PleD [22] with a few key differences (Figure S3C). The WspR crystal structure exists in a tetrameric assembly, with helical stalks from a C2-symmetry mate physically blocking the active sites of the diguanylate cyclase domains by being splayed apart (Figures 1D). We presume that the stalks have to extend the coiled-coil region in a WspR dimer to bring the GGDEF domains into close proximity for full cyclase activity (arrow in Figure S4A, center). This is in agreement with the prediction that the regions spanning residues 140 to 171 form a coiled-coil structure (Figure S1). Additionally, residues that comprise the coiled-coil contacts in the active dimer are identical to the residues involved in the antiparallel oligomerization of the stalks (Figure S4D–S4F).

### Cyclic di-GMP Binding Relies on Intact Active and Inhibitory Sites

**Figure 2:**
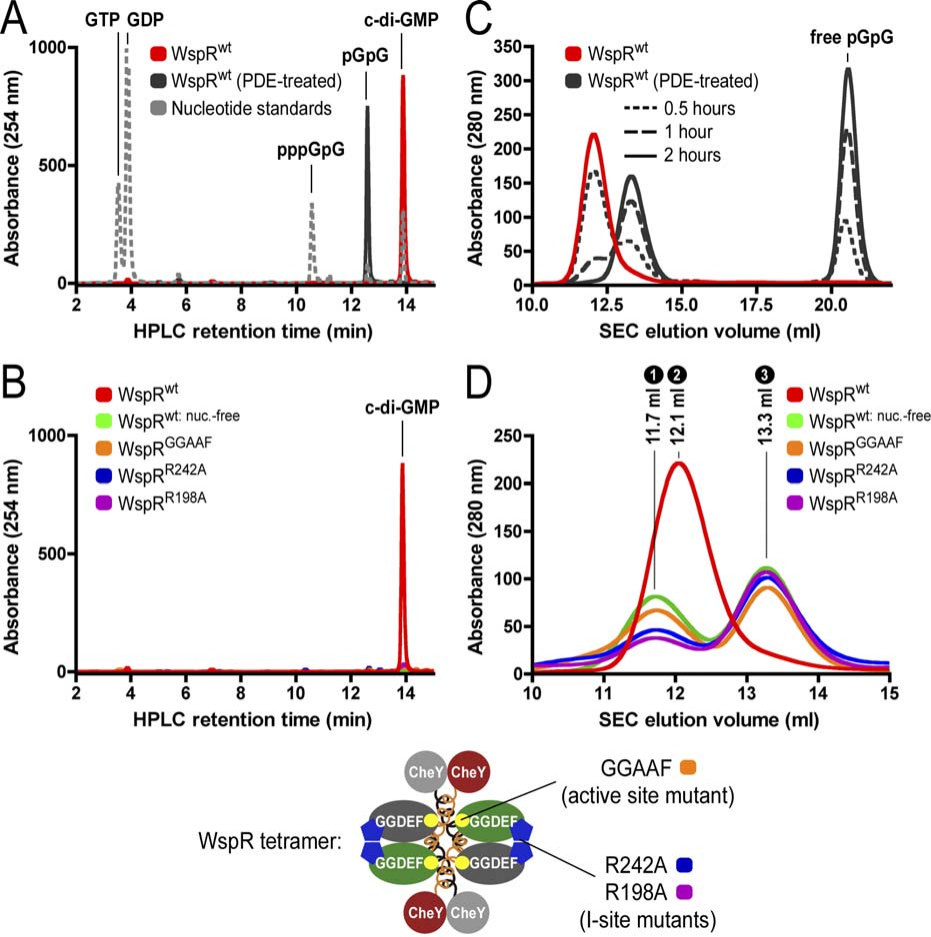
Cyclic di-GMP Binding and Gel Filtration Profile of Wild-Type and Mutant WspR (A) Detection of guanosine nucleotides by a reverse-phase HPLC-based assay. GTP, GDP, linear di-GMP (pGpG), c-di-GMP, and an intermediate condensation product (linear GTP-GMP; pppGpG) are well separated in this assay (grey dashed line). Products corresponding to pppGpG and pGpG were identified by mass spectrometry (unpublished data). Cyclic di-GMP was commercially available. WspR expressed in E. coli purifies with c-di-GMP bound (red trace) that is accessible for PDEs (black trace). (B) I-site and active site mutants of WspR purify nucleotide-free. Mutant proteins with disrupted I-sites (WspR^R242A^ or WspR^R198A^; blue and purple traces, respectively) or catalytic site (WspR^GGAAF^; orange trace) were analyzed. Nucleotide-free WspR^wt^ is obtained by PDE treatment followed by repurification using affinity and size exclusion columns (green trace). (C) PDE treatment triggers a conformational change in WspR. Cyclic di-GMP–bound WspR^wt^ (0.24 mM) was incubated with PDE (0.008 mM) in gel filtration buffer (25 mM Tris-Cl [pH 7.5], 100 mM NaCl, and 1 mM DTT) supplemented with 10 mM Mn^2+^ for 0.5, 1, or 2 h at 25 °C. Reactions were analyzed by SEC on a Superdex200 10/300 column (GE Heathcare). (D) SEC profiles of mutant and wild-type WspR. Nucleotide-bound and nucleotide-free WspR^wt^ (red and green traces, respectively), WspR^GGAAF^ (orange trace), WspR^R242A^ (blue trace), and WspR^R198A^ (purple trace) (0.24 mM) were analyzed by analytical gel filtration in gel filtration buffer. Peak maxima at 11.7 (peak 1), 12.1 (peak 2), and 13.3 ml (peak 3) are labeled. Peaks 1–3 (Superdex 200 10/30 column; GE Healthcare) correspond to peaks 1–3 (Shodex KW-803 column; JM Science, Inc.) in Table 1, obtained from the SEC coupled to the static multiangle light-scattering detectors.

As in the majority of diguanylate cyclases, WspR features a RxxD motif in the core of the I-site (R242xxD245 of WspR) spatially close to the active site's GGEEF motif [20] (Figure 1E). Similar to the structure of activated PleD, a crystal symmetry–related GGDEF domain complements the I-site of WspR by contributing a second arginine side chain (R198) [22]. To corroborate these structural observations, we investigated the identity of bound nucleotide to purified wild-type WspR (WspR^wt^; red trace) and mutant proteins. We used a high-performance liquid chromatography (HPLC)-based assay providing high-resolution separation and robust detection of guanosine nucleotides (Figure 2A). For the analysis, purified proteins were heat denatured, and filtered supernatants were subjected to reverse-phase chromatography.

**Table 1:**
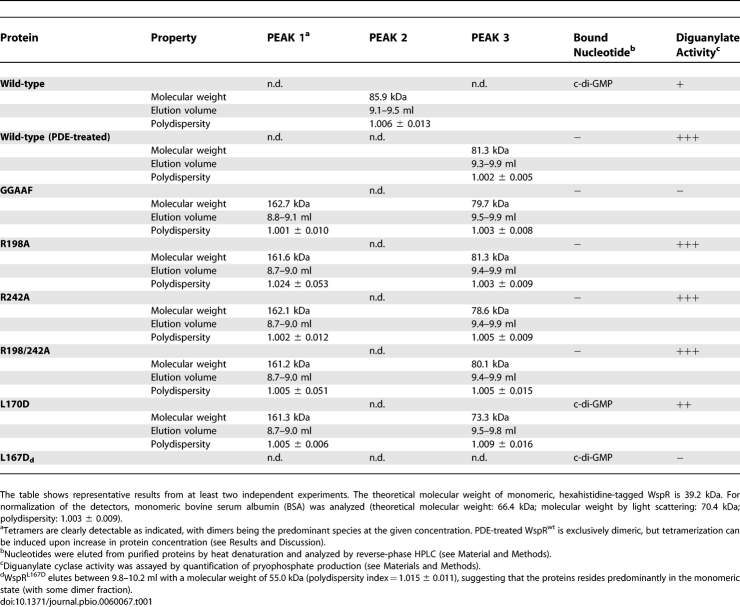
Molecular Weight of WspR Determined by Coupled SEC/Multiangle Light Scattering

Cyclic di-GMP co-purified with the enzyme upon overexpression in Escherichia coli (Figure 2A). The intrinsic off-rate of nucleotide from the I-site was extremely slow with no significant loss of c-di-GMP observed upon dialysis for several days (unpublished data). To produce a nucleotide-free species of WspR, we preincubated WspR^wt^ with an EAL domain–containing PDE, SadR/RocR from P. aeruginosa. Similar results were obtained with other c-di-GMP–specific PDEs, suggesting that WspR-bound c-di-GMP is readily accessible for catalytic cleavage (unpublished data). PDE treatment results in the degradation of c-di-GMP to free pGpG that could be easily removed by gel filtration (Figure 2A and 2C). We distinguish between PDE-treated (WspR^wt: PDE-treated^; black trace) and nucleotide-free (WspR^wt: nuc.-free^; green trace) WspR^wt^. In the latter case, PDE treatment was part of the purification protocol, but PDE and free nucleotides had been removed. WspR^wt: nuc.-free^ purification proceeded as described for WspR^wt^ and mutant proteins (see Material and Methods).

A catalytically dead mutant in which the two central glutamate residues of the active site have been replaced by alanines (WspR^GGAAF^; orange trace) purified nucleotide-free (Figure 2B), indicating that WspR's activity was required for c-di-GMP binding to the I-site in cells. Yet, purified WspR^GGAAF^ was able to bind c-di-GMP when incubated with free c-di-GMP (unpublished data). Site-directed mutants that lack either the R242 or R198 side chains at the I-site (WspR^R242A^ and WspR^R198A^; blue and purple trace, respectively) purified essentially nucleotide-free but are highly active enzymes (Figures 2B) (see below), suggesting that c-di-GMP has low affinity for the active site.

### Cyclic di-GMP Stabilizes WspR in an Elongated, Dimeric Conformation

Degradation of c-di-GMP by PDE treatment has a profound effect on the elution profile of WspR in size-exclusion chromatography (SEC) (Figure 2C). Nucleotide-bound WspR^wt^ eluted at approximately 12.1 ml. Upon incubation with PDE (30:1 molar ratio of WspR to PDE), this peak decreased, and a new peak appeared at an elution volume of approximately 13.3 ml, with complete conversion after 2 h of incubation. In the nucleotide-bound enzyme, c-di-GMP contributes significantly to the absorbance at 280 nm. Release of pGpG, the product of the PDE-catalyzed reaction, led to a decrease of absorbance across the protein-containing peak in parallel with the appearance of a nucleotide peak coeluting with other small molecules and salts (Figure 2C).

**Figure 5:**
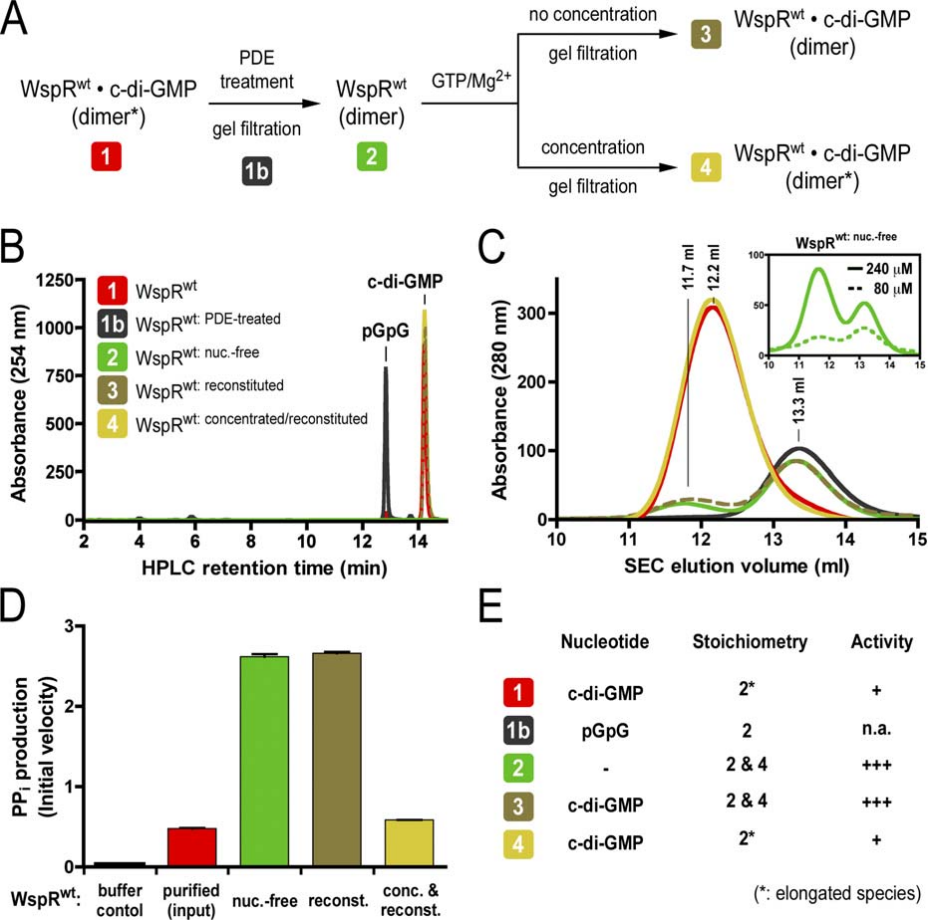
Reconstitution of a Product-Inhibited WspR (A) Flow chart outlining the reconstitution experiments. Cyclic di-GMP–bound, inhibited WspR^wt^ (sample 1) (0.24 mM) was treated with PDE (sample 1b). The nucleotide-free, active dimer was repurified (sample 2). After incubation with GTP/Mg^2+^, the sample was split into two fractions. One fraction was concentrated (to approximately 0.48 mM) and incubated overnight at 4 °C, the other was incubated under identical conditions at its initial concentration (approximately 0.05 mM) (samples 3 and 4, respectively). Samples were subjected to SEC. At each step of the reaction cycle, samples were analyzed for nucleotide content, their gel filtration profile, and enzymatic activity. (B) Nucleotide loading states of WspR species. Proteins were analyzed as described above in Figure 2. (C) Gel filtration profiles of WspR species. WspR^wt^ species indicated in Figure 5A were analyzed as described above (Figure 2C and 2D). Except for the sample that was subjected to a final concentration (sample 4), the concentration of WspR (initially at 0.24 mM) decreased along the reaction scheme due to preparative gel filtration steps. The inset shows concentration-dependent oligomerization behavior of nucleotide-free WspR. WspR^wt: nuc.-free^ (dashed line: 0.08 mM; solid line: 0.24 mM; initial concentration) was analyzed by SEC. Peak maxima are at 11.7 and 13.3 ml elution volume. (D) Enzymatic activity of WspR species. Catalytic activities of WspR species (0.5 μM) were determined in a continuous assay measuring pyrophosphate production as described in Figure 4B. Initial velocities were determined by linear regression. Error bars indicate standard deviations of three independent experiments. (E) Structural and functional characteristics of distinct WspR states. The table summarizes properties of distinct WspR species along the reconstitution path.

A similar analysis was applied to nucleotide-free WspR^wt^ and mutants with disrupted active (WspR^GGAAF^) or I-site (WspR^R242A^ or WspR^R198A^) (Figure 2D). Whereas c-di-GMP–bound WspR^wt^ eluted as a single peak (peak 2 in Figure 2D), the nucleotide-free proteins showed a bimodal distribution with peaks at an elution volume of approximately 11.7 ml (peak 1 in Figure 2D) and, similar to the PDE-treated sample, of approximately 13.3 ml (peak 3 in Figure 2D). The relative distribution was dependent on protein concentration with a shift to faster eluting proteins at higher concentration (see below; Figure 5C). The detection of these different species by gel filtration indicated the existence of distinct conformations of WspR depending on its nucleotide-bound state.

**Figure 4:**
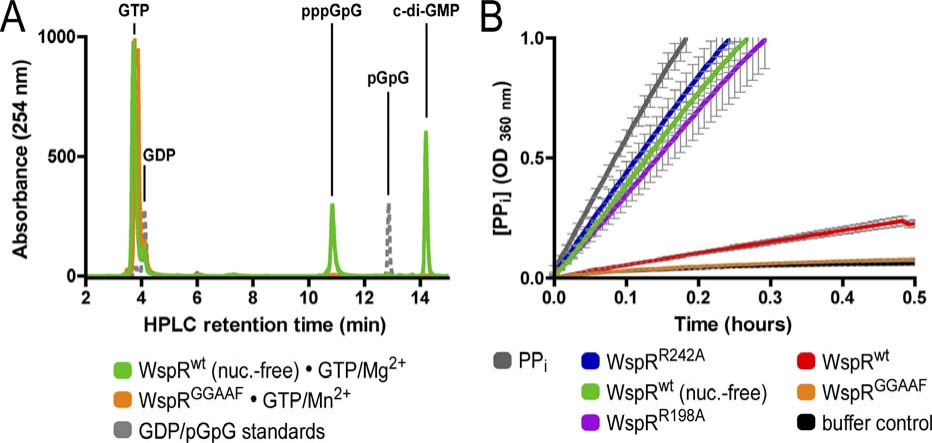
Activity of Distinct WspR Species (A) HPLC-based activity assay measuring c-di-GMP production by WspR^wt^. Nucleotide-free WspR^wt^ (green trace) or WspR^GGAAF^ (orange trace) (10 μM) was incubated in buffer containing GTP/Mg^2+^ (1 mM/2 mM) for 1 h at 25 °C. Nucleotides were analyzed by reverse-phase HPLC after heat denaturation of proteins and ultrafiltration of the supernatants, and reaction products were compared to retention times of well-characterized nucleotide standards (grey trace and Figure 2A). (B) Comparison of enzymatic activity of wild-type and mutant forms of WspR. WspR^wt^ (nucleotide-bound or -free), or mutant variants (0.5 μM) were incubated at 25 °C in assay buffer (EnzChek Pyrophosphate Assay; Invitrogen) containing GTP/Mg^2+^ (0.5 mM/2 mM), and pyrophosphate production was measured by continuously monitoring absorbance at 360 nm. Coloring corresponds to the scheme in Figure 2. Error bars indicate standard deviations of three independent experiments. Incubation of inorganic pyrophosphate (PP_i_; grey trace) (0.5 mM) in assay buffer determines the rate limit of the assay system. In the buffer control (black trace), GTP/Mg^2+^ is included in the reaction.

SEC has the limitation that it is not possible to distinguish whether an observed shift in elution is due to a conformational change affecting the shape of the particle (e.g., rod-shaped versus globular) and/or a change in stoichiometry, an obstacle for calibration of this method. In order to determine the absolute molecular weight, and hence assembly state of WspR in solution, we turned to static multiangle light scattering (coupled to SEC) [30]. The technique relies on the fact that the intensity of scattered light produced by a macromolecule is proportional to its molecular weight. Sample concentration and time-averaged intensity of scattered light, collected simultaneously at different angles, are measured as the protein elutes from a gel filtration column. The measurements are insensitive to the shape of the molecules and assemblies, yielding the absolute molecular weight and polydispersity indices that provide an estimate for the mass distribution in the sample [31]. In addition, molecular weights can be correlated to the elution time in SEC.

**Figure 3:**
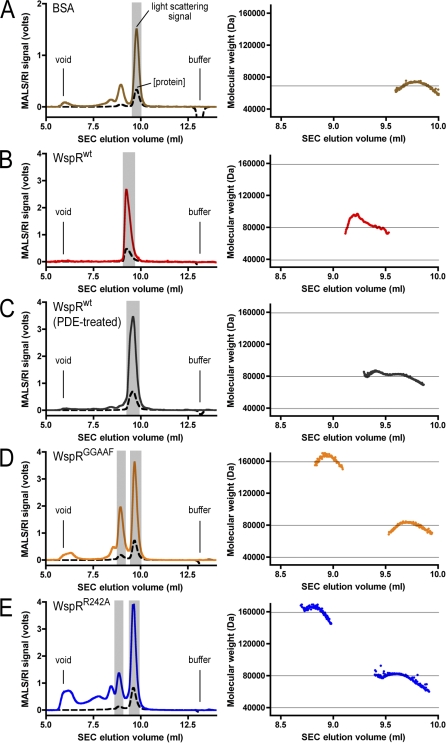
SEC-Coupled Multiangle Light-Scattering Analysis of Purified WspR in Solution (A) Monomeric BSA (4 mg/ml; Sigma) was analyzed by coupled SEC/multiangle light scattering. The mobile phase consists of 25 mM Tris-HCl (pH 7.5), 100 mM NaCl, and 1 mM DTT. In the left panel, the primary signal (in volts) is plotted against the elution volume. The solid (colored) trace shows the signal of one of the light-scattering detectors (at 90° to the incident beam). The signal from the refractive index detector is shown as a dashed line. The grey area highlights the analyzed peak. The void volume (void) and the end of the experiment (buffer) are indicated. In the right panel, molecular weights, determined by light scattering, and protein concentration, measured by change of refractive index, from each data slice (0.5-s increments) are plotted against the elution volume. The grey horizontal line indicates the theoretical molecular weight. (B–E) Cyclic di-GMP–bound WspR^wt^ (4 mg/ml) (B), PDE-treated WspR^wt^ (4 mg/ml) (C), WspR^GGAAF^ (4 mg/ml) (D), and WspR^R242A^ (4 mg/ml) (E) were analyzed as described in (A). The grey horizontal lines indicate the theoretical molecular weight for monomers, dimers, and tetramers.

The results for mutant and wild-type proteins discussed above are shown in Figure 3 and summarized in Table 1 (including nucleotide binding and activity data). Bovine serum albumin (BSA) served as an isotropically scattering sample for the normalization of the light-scattering detectors (Figure 3A). The averaged molecular weight measured across the peak corresponding to monomeric BSA (70.4 kDa, with a polydispersity index of 1.003) is in good agreement with the theoretical value (66.4 kDa).

To our surprise, both nucleotide-bound and PDE-treated WspR were essentially dimeric at a concentration of approximately 4 mg/ml (0.1 mM; initial concentration before gel filtration) but eluted at distinct positions, as observed with gel filtration (Figure 3B and 3C; Table 1). Given identical stoichiometry, both states differ only in their hydrodynamic radius with the c-di-GMP–bound WspR^wt^ eluting as an elongated dimer, whereas PDE-treated WspR appears to be more globular in shape. Low polydispersity indices and the absence of tetramers in these samples indicate that dimers were the predominant species under these conditions and that conversion from the elongated to compact species might be direct, not transitioning through a tetrameric state (Table 1). Alternatively, the tetramer might be a very transient intermediate during the switching between the dimeric states. A small, but detectable, tetrameric fraction assembled upon prolonged incubation in the absence of c-di-GMP (see below; Figure 5C).

In contrast, the nucleotide-free mutants (WspR^GGAAF^, WspR^R242A^, or WspR^R198A^) partitioned into a compact dimer and a tetrameric species eluting later and earlier, respectively, than the elongated c-di-GMP–bound WspR^wt^ dimer (Figure 3D and 3E; Table 1). Since tetramerization occurred in the nucleotide-free proteins with disrupted I-site or active site, higher-order oligomerization is likely to be driven by interactions of the stalk motifs rather than c-di-GMP.

### Elongated and Compact Dimers Exhibit Distinct Catalytic Activities

To assess whether the different oligomeric species correspond to a certain activity state, we applied both an HPLC-based and a colorimetric assay. Reaction products from incubations of nucleotide-free WspR^wt^ with GTP/Mg^2+^ were analyzed (Figure 4A). In addition to a GTP peak eluting at 3.9 min, the HPLC profile showed two peaks eluting at 10.8 and 14.2 min, respectively. The peak with the longer retention time corresponds to c-di-GMP based on its coelution with purified c-di-GMP (Figures 4A and 2A). Mass spectroscopy was used to identify the other peak as a product resulting from a single phosphodiesterification of two GTP molecules to a linear GTP-GMP species (pppGpG). Purified pppGpG could serve as a substrate for WspR resulting in the production of c-di-GMP, suggesting that it is an intermediate along the reaction coordinates for diguanylate cyclization (unpublished data). Addition of BeF_3_^−^ or acetyl phosphate did not accelerate the reactions (unpublished data), consistent with constitutive dimerization of WspR even in the absence of phosphorylation (Table 1; see Materials and Methods).

For kinetic analysis of wild-type and mutant WspR, we turned to a coupled enzyme assay that monitors the production of pyrophosphate, a product of the cyclization reaction. Whereas WspR^GGAAF^ was catalytically inactive, showing no activity above background (buffer control), all other compact, dimeric species of WspR (nucleotide-free WspR^R242A^, WspR^R198A^, WspR^wt: nuc.-free^, and c-di-GMP–bound WspR^L170D^) were highly active (Figures 4B and S6). In contrast, the elongated, nucleotide-bound WspR^wt^ dimer showed slow kinetics. In light of the model discussed below, the residual activity most likely originated from a small fraction of an active species of the protein. Thus, the compact and the elongated dimeric conformations of WspR correspond to distinct active and inactive states of the enzyme.

The residues involved in oligomerization, c-di-GMP binding, and production are conserved in WspR from other *Pseudomonas* species (Figure S5A). In order to analyze conservation of the regulatory mechanism, we cloned, expressed, and purified WspR from P. putida, *fluorescence*, and *syringae*. The protein sequences are 74%–84% identical to each other (including P. aeruginosa) (Figure S5B). The results obtained for these proteins were equivalent to the ones described above for WspR from P. aeruginosa with regards to nucleotide binding state, switching mechanism, and activity (Figure S5C–S5E).

We also analyzed mutant enzymes with aspartate substitutions in the hydrophobic residues critical for coiled-coil formation and oligomerization (WspR^L170D^ and WspR^L167D^) (Figure S4D–S4F). Results for oligomerization propensity, nucleotide-bound state, and activity are summarized in Table 1 and Figure S6. Whereas WspR^L170D^ existed in a compact dimer–tetramer equilibrium similar to the one observed for nucleotide-free forms of WspR, WspR^L167D^ eluted later, with a molecular weight measured to be 55.0 kDa, suggesting a weakly associating dimeric species (Figure S6C and Table 1). Both WspR^L170D^ and WspR^L167D^ purified with c-di-GMP bound (Figure S6B), but only WspR^L170D^ had significant catalytic activity (Figure S6D). WspR^L167D^ was inactive in the conditions used. PDE treatment of WspR^L170D^ and WspR^L167D^ had no effect on their catalytic activity (unpublished data), suggesting that c-di-GMP binding to the I-site is not sufficient for achieving product inhibition (see below). Taken together, these results highlight the importance of dimerization driven by the CheY domains and the protruding coiled-coil motifs for activity and the importance of the leucine residues in establishing the autoinhibited state.

### Tetramerization Is an Essential Step in Establishing Feedback Inhibition

To investigate the molecular mechanism for product inhibition, we reconstituted a reaction cycle starting with the elongated, c-di-GMP–bound inactive WspR^wt^ species (dimer*; red trace; Figure 5A). For each step in the reaction cycle outlined in Figure 5A, we determined the nucleotide-bound state, analytical gel filtration profile (providing insight into molecular weight and shape of the protein), and diguanylate cyclase activity (Figure 5B–5E).

As observed before, treatment of WspR with PDE produced pGpG, removing the inhibitor from the I-site (Figure 5B). This reaction was accompanied by a conformational change altering the mobility of WspR^wt^ to a later eluting, more compact species (sample 1b; WspR^wt: PDE-treated^; black trace) when compared to sample 1, the original elongated dimer (Figure 5C). PDE and free nucleotide were removed, and nucleotide-free WspR was subjected to gel filtration, following the protocol for the preparation of WspR^wt: nuc.-free^ (see above; Material and Methods). Over the course of this treatment, a small fraction of the tetrameric species, eluting at 11.7 ml, assembled although the enzyme concentration was not altered significantly (sample 2; WspR^wt: nuc.-free^; green trace) (Figure 5C).

Cyclic di-GMP production was initiated by the addition of GTP and Mg^2+^. At this point, half of the sample was concentrated (leading towards sample 4) and incubated in the presence of c-di-GMP overnight. The other sample was incubated similarly without varying its concentration (leading towards sample 3). Excess nucleotide was removed by SEC, and peak fractions were analyzed by analytical gel filtration and HPLC analysis (Figure 5B and 5C). The mobility of the sample that had not been subjected to a concentration step (sample 3; olive trace) was unchanged compared to the nucleotide-free species despite c-di-GMP binding to the I-site. In contrast, c-di-GMP–bound WspR^wt^ that was incubated at higher protein concentration in the presence of nucleotide (sample 4; yellow trace) eluted at the same position as the original enzyme (sample 1; red trace), reminiscent of an elongated, product-inhibited dimer.

When comparing enzymatic activities associated with the distinct reaction states, we found that the product-inhibited species was only reconstituted in the case where the protein concentration was raised and thus facilitating tetramer formation (sample 4) (Figure 5D and inset in Figure 5C). Despite c-di-GMP binding, the form of WspR^wt^ existing predominantly as a compact dimer (sample 3) was highly active similar to the nucleotide-free species, consistent with the identical elution profiles (Figure 5D and 5E). Both conditions, favoring tetramer formation and the presence of c-di-GMP, were required for assembling the elongated, inactive WspR dimer. We hypothesize that the tetramer serves as a platform allowing the formation of the product-inhibited state, and c-di-GMP serves as a coordinator as well as an allosteric inhibitor in the final conformation (see below).

### Expression Levels of WspR Determine Its Enzymatic Activity in Cells

**Figure 6:**
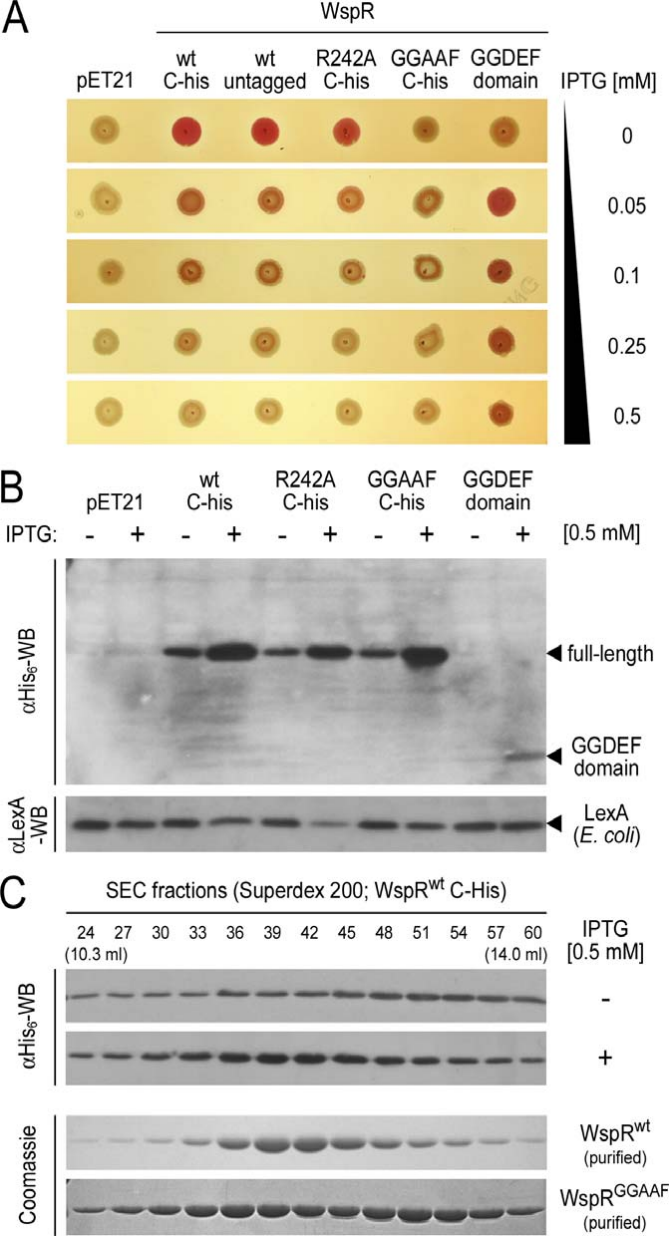
Catalytic Activity and Oligomerization of WspR in Cells (A) Congo Red (CR) assay monitoring WspR-catalyzed c-di-GMP production in cells. E. coli BL21 were transformed with plasmids encoding wild-type or mutant variants of WspR. Cells were grown to mid-log phase at 37 °C, and 2.5 μl of the culture was spotted onto a CR-containing LB plate with or without IPTG and incubated for 24 h at 30 °C. Leaky expression in the absence of IPTG and IPTG-induced WspR expression was visually assayed for a red colony phenotype (rdar morphotype). Cells expressing an untagged version of WspR^wt^ behave similarly to cells expressing WspR with a C-terminal hexahistidine tag. (B) Loss of CR staining correlates with high WspR expression levels. Cultures were grown for 16 h at 25 °C in the absence or presence of IPTG. Lysates from cells expressing hexahistidine-tagged wild-type and mutant variants of WspR (see above) were prepared by sonication and analyzed by western blotting using a hexahistidine tag-specific antibody to detect recombinant protein. Samples were normalized to total protein amount prior to SDS-PAGE and blotting. Western blot detection of the native E. coli protein LexA was used as a control. LexA levels were slightly lower in some samples due to high WspR expression levels obscuring total protein normalization. (C) Gel filtration profile of WspR in cell lysates. Cell lysates were subjected to SEC in gel filtration buffer. Fractions (0.1 ml) were collected, and hexahistidine-tagged proteins in the fractions were detected by western blotting. Elution profiles were compared to profiles obtained for purified proteins (c-di-GMP–bound WsrR^wt^ or nucleotide-free WspR^GGAAF^) under identical conditions.

All results discussed thus far have been obtained using a purified system presenting evidence for a mechanism of how WspR might integrate enzyme and product concentration to modulate its activity state. To corroborate these findings in cells, we adopted a phenotypic assay that correlates c-di-GMP production with increased Congo Red (CR) staining characteristic for cellulose production in E. coli [32,33]. E. coli BL21 transformed with the vector control (pET21) or WspR^GGAAF^ remained uncolored when plated on CR-containing plates (Figure 6A). In contrast, leaky expression of WspR^wt^ or WspR^R242A^ in the absence of IPTG was sufficient to cause a red colony phenotype (Figure 6A and 6B). Increased IPTG-induced protein expression impaired the morphotype, and colonies remained uncolored. In contrast, the isolated GGDEF domain was inactive at low expression levels, but CR staining was detectable upon induction with IPTG, suggesting that bulk protein expression is sufficient for the formation of transient functional complexes.

Based on the analysis by gel filtration of lysates from E. coli expressing WspR^wt^ at low and high levels, it was evident that an increase in protein expression was accompanied by a shift in hydrodynamic radius of WspR^wt^. Upon IPTG induction, WspR^wt^ shifted from a compact dimer at low expression to a species with a peak eluting at a similar position as the purified c-di-GMP–bound elongated dimer (Figure 6C). The elution behavior was distinct from that of purified WspR^GGAAF^ that eluted as a tetramer-compact dimer mixture (Figure 6C and Table 1). We hypothesize that WspR^R242A^ forms tetramers at high protein concentrations in cells in which the active sites are blocked, therefore inhibiting the morphotypic changes. Gel filtration experiments starting with lysates from WspR^R242A^- or WspR^GGAAF^-expressing cells showed predominantly the more compact, dimeric species, probably due to the dilution occurring during chromatographic separation (unpublished data).

Taken together, the basic mechanism of product inhibition and conformational switching of WspR derived from the structural and functional studies appears to be effective in cells.

### Model for the Inhibition and Activation of WspR

**Figure 7:**
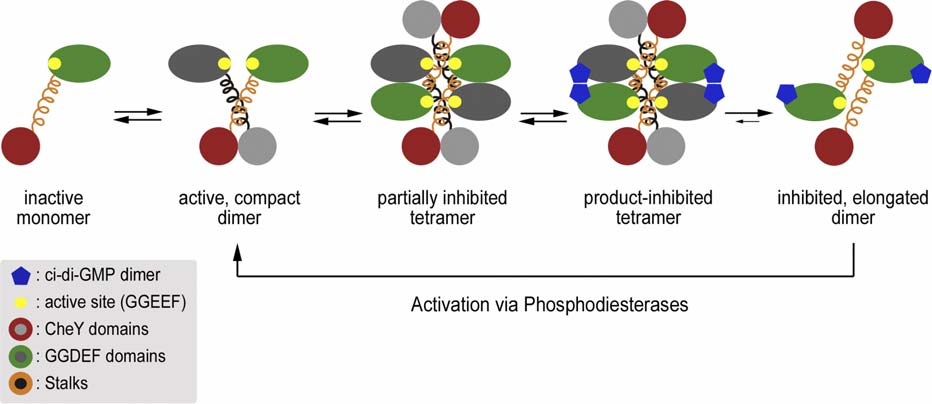
Model for the Feedback Regulation and Reactivation of WspR Based on structural and functional analyses, a model is proposed in which active WspR dimers are in equilibrium with a transient tetrameric species that, in the presence of c-di-GMP, provides an exit platform for the product-inhibited elongated dimer. Degradation of c-di-GMP by PDEs triggers a snapping back to the active dimer species, probably avoiding the tetrameric state. Both c-di-GMP binding and tetramerization are required for the assembly of the inhibited species. It is unlikely that the tetrameric state is en route from the inhibited to the active dimer, which would require dissociation of the tetramer once c-di-GMP has been degraded. Experimentally, we did not observe any tetramers immediately after PDE treatment, and the tetrameric species arises only after prolonged incubation of the compact, but not the elongated dimer (Table 1, see also Figures 2C, 3, and 5).

**Figure 8:**
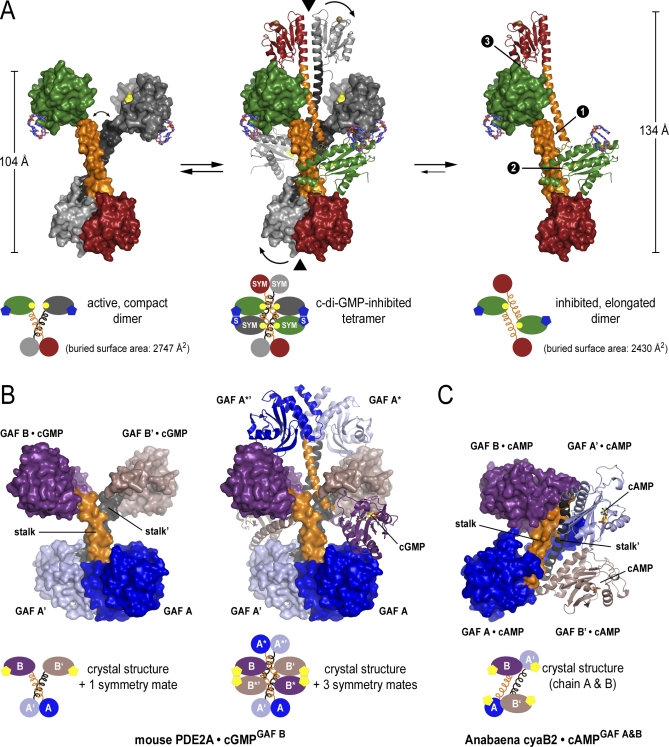
Distinct Oligomeric Conformations of WspR and Mechanistic Implication for the Regulation of GAF Domain-Containing Proteins (A) Structural models closely resembling the distinct states of WspR. The solvent-accessible surface of a WspR dimer is shown. In the active state (left panel), dimerization is mediated by the CheY-homology domains and protruding stalks. The stalks may convene in the fully active state bringing the GGDEF domains into close proximity (arrow in left panel). In the tetrameric assembly that serves as an intermediate between the two dimeric states (middle panel), two C2-symmetry–related crystallographic dimers of WspR are shown intertwined in a head-to-head orientation. “S” or “SYM” in the cartoon diagram indicates crystal symmetry–related molecules. The stalks form a tetrameric structure splaying apart the coiled-coils and physically blocking the active sites. Cyclic di-GMP molecules bound at the I-site bridge the GGDEF domains of neighboring molecules. Arrows indicate how breaking up the CheY domain dimer by a rigid body rotation of the CheY-stalk module would facilitate the formation of two identical dimers shown in the right panel. In the proposed model for the product-inhibited state, dimers of two symmetry-related molecules (chain A) are held together by three interfaces between the tip of the stalks (zone 1), between the GGDEF domain and the stalk (zone 2), and between the GGDEF domain and the CheY domain (zone 3) of adjacent molecules. Such dimers can be readily obtained from the tetramer by segregation of a plane consisting of the colored molecules (chain A dimer) from a plane formed by the grey molecules (chain B dimer). The maximal dimensions and surface areas buried at the interfaces are shown. (B) Dimeric and tetrameric assembly seen in the mouse PDE2A tandem GAF domain crystal structure. The molecule in the asymmetric unit and a symmetry-related molecule (indicated by apostrophes) form a dimer via pairing of their GAF A domains (PDB code 1MC0) [39]. In the crystal, the tips of the stalks that connect the GAF A and GAF B domains are splayed apart by a symmetry-related dimer (see tetrameric structure). Cyclic GMP is bound only to the GAF B domains. The tetrameric assembly consists of two additional symmetry mates (indicated by asterisks). The two symmetry-related crystallographic dimers are intertwined in a head-to-head orientation with the stalks forming a tetrameric structure via their split ends. In such an assembly, significant interfaces for the dimer–dimer interaction are formed between the stalks, between the GAF B domains, and between the GAF A and B domains of adjacent chains. GAF A domains are colored blue and light blue, GAF B domains are colored violet and light violet, and the stalks are shown in orange and grey. (C) Crystal structure of the tandem GAF domain dimer from the adenylate cyclase cyaB2 in *Anabaena*. The two tandem GAF domain molecules in the asymmetric unit form an antiparallel dimer mediated in part by the helical stalks connecting the GAF A and B domains (PDB code 1YKD) [38]. Cyclic AMP is bound to both GAF A and B domains of cyaB2. The coloring scheme in (B) has been applied for straightforward comparison of relative domain orientations.

The gel filtration and multiangle light-scattering experiments demonstrated that in solution, c-di-GMP-inhibited WspR is predominantly an elongated dimer, distinguishable from an active dimer with a more globular shape and from a tetrameric species (Figure 3). The reconstitution experiment showed that the tetramer serves as a scaffold that assembles from the active, compact dimer and dissociates into the elongated, inhibited dimer (Figures 7 and 8A). The crystallographic tetramer assembled from this elongated, dimeric WspR, and hence, we hypothesize that the model contains structural information about both dimeric states. We already discussed a suggestive active dimer conformation (see above; Figure 1B) and will now propose a model for the elongated dimeric state.

Although three possible elongated WspR dimers can be derived from the tetrameric structure (Figure S4C), the dimer shown in Figure 8A (right panel) might represent a structure close to the conformation of inactive WspR in solution (elongated dimer 1 in Figure S4C), based on structural and functional arguments. A detailed rationale is provided as supplemental information (Figure S3C, figure legend), and we will concentrate on the description of the mechanistically most plausible model (Figure 8A).

There are many considerations that support elongated dimer 1 as the inhibited state (Figures 8A and S4C): Structurally, such a dimer can be readily obtained from the tetrameric assembly by a dissociation of the two planes consisting of the colored (chain A) and the grey (chain B) molecules, respectively (Figure 8A). Such segregation would be accompanied by the breakup of the CheY dimer. This would require a simple rigid body rotation of the stalk-CheY unit in the dimer shown in grey (arrows in Figure 8A, middle panel) that is similar to the rotation shown in Figure 1C. The breakup of the CheY dimer interface would be thermodynamically facilitated by new interactions formed between the GGDEF domain and the CheY-stalk unit of neighboring molecules (zone 2 and 3 in Figure 8A, right panel). The proposed dimer interface is the most extensive one of the three possible dimer states (∼2,430 Å^2^ surface area buried; Figure S4C) and is spread over the entire molecule, including an antiparallel packing of the stalks, in addition to the aforementioned interactions (zones 1–3 in Figure 8A, right panel). The GGDEF-stalk interface (zone 2) is centered around an ionic bond between residues E253 from the active site GGEEF motif and R151 in the stalk of an adjacent protomer (Figure S4C, inset). In addition to stabilizing the antiparallel assembly, this interaction would contribute to the inhibition mechanism by sequestering the active site, with c-di-GMP bound at the back of the β-sheets connecting the GGEEF motif-containing loop serving as an allosteric inhibitor, in agreement with a model for c-di-GMP–mediated inhibition proposed recently [20]. Altogether, such a conformation would leave the CheY-homology domains unpaired and available for dimerization, whereas the GGDEF domains are far apart from each other in a catalytically incompetent state.

The linkage between the GGDEF domain and the CheY-stalk module is likely to be rather flexible, as evidenced by the differences observed between the two molecules in the asymmetric unit (Figure 1C). For both the models of the active and inactive dimers, the exact position of the GGDEF domains relative to the CheY-stalk module might be different from that observed in the tetrameric assembly, and might depend on interactions with nucleotides, inter- and/or intramolecular interactions. Further studies will be needed to determine the exact conformations of these states.

### Conclusion

The diguanylate cyclase WspR, a response receiver that controls biofilm formation in *Pseudomonas*, is subject to a surprisingly sophisticated mode of regulation, considering its rather straightforward domain organization. The autoregulatory switch in WspR involves a coiled-coil motif extending from the CheY-homology phospho-receiver domains. This helical stalk can homodimerize in a parallel or antiparallel fashion, stabilizing the active or inactive state, respectively (Figure 8A). Since the tetramer assembles from the compact dimeric species and dissociates into elongated dimers in the presence of c-di-GMP, it is not surprising that the structure captures features from all three states. As a required intermediate, a tetrameric assembly serves as an exit platform for the product-inhibited species. Tetramerization is also driven by interactions of the stalks, jamming the active sites and establishing transient inhibition.

We recently obtained a crystal structure of the WspR homolog from P. syringae (R. Raghavan and H. Sondermann, unpublished data). In this crystal, WspR was also found in a tetrameric assembly similar to the one described here with a subtle, but important, difference. The inhibitory contacts between all four GGDEF domains and the CheY-stalk modules of the neighboring protomers have been formed (similar to zone 2 in Figure 8A). It was rewarding to see that this leads to a weakening of the CheY domain interfaces (unpublished data), supporting our hypothesis that the tetramer facilitates the formation of elongated, inactive dimers with unpaired CheY domains.

Previously, it has been demonstrated that WspR is regulated by phosphorylation in vivo [23,24,34]. In the purified system, mutant proteins targeting the active site of the CheY domain have characteristics indistinguishable from WspR^wt^ (unpublished data). Furthermore, purified WspR appears to be constitutively oligomeric even in the absence of posttranslational modifications under the conditions described here (Table 1; Materials and Methods). Constitutive, phosphorylation-independent dimerization has been shown recently for HP-RR, a response regulator from Helicobacter pylori [35], and might apply more widely to other CheY-homology domain–containing proteins.

Although the compact, active dimer state and the regulatory mechanism appear not to require phosphorylation of the CheY domains, we do not rule out that it might have an effect on WspR function. In fact, preliminary studies suggest that addition of beryllium fluoride, a commonly used compound mimicking phosphorylation, modulates WspR's oligomeric state, facilitating tetramer formation and autoinhibition (N. De and H. Sondermann, unpublished data). In a putative model, the activated, phosphorylated state may be subject to stronger feedback regulation via tetramerization. Such a mechanism would create a short c-di-GMP pulse before WspR shuts down contributing to localized signaling, a hallmark described for this second messenger signaling [36]. On another level, WspR has been shown to relocalize in a phosphorylation-dependent manner [37]. It is feasible that phosphorylation is used for fine-tuning of the signaling system. The proper output might rely on the exact spatial and temporal activation of WspR and regulation might impact both localization and/or activity. The exact mechanism and role of phosphorylation on WspR activity awaits further experimental elucidation.

The switching mechanism in WspR involving distinct oligomers might be more widely used, and may apply to other regulatory domains such as GAF domains, a common regulatory module in cyclic nucleotide PDEs. Tandem GAF units contain two globular nucleotide-binding domains connected by a helical stalk. They have been crystallized in two conformations, a parallel and an antiparallel dimer [38,39]. In the structure of mouse PDE2A, the N-terminal GAF domain and the protruding stalk function as a dimerization module (Figure 8B, left panel) [39]. Interestingly, we find a tetrameric assembly in the crystal lattice in which the stalk dimers and the second GAF domains bind in a head-to-head orientation (Figure 8B, right panel). In essence, these conformations show remarkable resemblance to the compact dimer and the tetramer species of WspR (Figure 8). In contrast, the tandem GAF domain from *Anabaena* forms an antiparallel assembly in the crystal lattice (Figure 8C) [38], globally resembling the elongated dimer of WspR (Figure 8). We speculate whether a similar oligomerization mechanism as the one we describe here for WspR might also regulate GAF domain–containing phosphodiesterases in all kingdoms of life.

In our model for feedback regulation of WspR, c-di-GMP serves two purposes: Initially, it works as a clamp in the tetramer that stabilizes a conformation from which the inactive dimers detach. Cyclic di-GMP remains bound to the canonical I-site motif located solely at the GGDEF domain, where it functions as an allosteric inhibitor [20]. The inhibited dimeric state is primed for (re)activation. Removal of c-di-GMP from the I-site by PDEs releases the inhibition and induces dimerization of the unpaired CheY-homology domains and diguanylate cyclase activity via switching between distinct dimer species. Using purified enzymes, we did not observe a high degree of specificity; multiple active PDEs showed similar activities towards WspR-bound c-di-GMP. In cells, targeting and signaling specificity might be determined by regulatory domains in the PDEs. In summary, activation by PDEs might represent a more general way to switch on diguanylate cyclases, in addition to the well-established phosphorylation-dependent mode.

The feedback mechanism described here allows WspR to integrate multiple inputs such as phosphorylation, as well as protein and c-di-GMP concentrations, to adjust its activity. The mode of regulation is likely to be relevant with regards to the particular properties of c-di-GMP signaling in bacteria. Despite its small size and hydrophilic nature, c-di-GMP appears not to function as a general, diffusive second messenger in the cell. Mutation and overexpression of individual diguanylate cyclases and phosphodiesterases in *Pseudomonas* lead to distinct cellular responses for the particular enzymes, affecting either biofilm formation, type III-secretion system-mediated cytotoxicity, or both [17]. Rather than triggering pleiotropic signaling, responses appear to be spatially restricted, probably depending on the localization of the enzymes producing or turning over c-di-GMP [36,40,41]. Both displaying an inhibitory site with high affinity for c-di-GMP on the cyclases themselves, and mechanisms for sensing and reacting to the local enzyme concentration can contribute to spatial restriction of the signal.

All residues involved in higher-order oligomerization, c-di-GMP binding, and catalysis are strictly conserved across various *Pseudomonas* strains (Figure S1). It is interesting to note that a subset of *Burkholderia* (*Burkholderia sp. 383*, *ambifaria MC40–6*, and *vietnamiensis G4*) contains a point mutation in a residue critical for c-di-GMP binding at the I-site (R242; Figures S1 and 1E). These *Burkholderia* species are frequently found in cystic fibrosis patients with pulmonary infections [42]. It will be interesting to see whether the substitution in the I-site of WspR and c-di-GMP signaling in general contribute to virulence mechanism of microorganisms. More extensive analyses of the genomes from clinical isolates, in combination with thorough mechanistic studies, will be required to establish a link of pathogenicity and altered activities of enzymes involved in c-di-GMP signaling.

Many processes, including bacterial biofilm formation, contribute to the pathogenicity of microorganisms and high mortality rate from infectious diseases [43]. Biofilms have been shown to be the leading cause for chronic infections such as Legionnaire's disease, infections of the heart and ear, and infections accompanied with cystic fibrosis, to name only a few prominent examples [44–46]. Often, bacterial biofilms escape efficient treatment due to the development of tolerance or resistance to antibiotics [15]. Identifying the molecules and mode of action contributing to pathogenesis, such as c-di-GMP and enzymes for its production and turnover, provides the foundation for novel strategies in the treatment of infections [17,47]. Given the absence of c-di-GMP signaling in eukaryotic cells, this pathway might be an attractive therapeutic target and mechanistic studies will facilitate the development of novel antibiotics in this regard.

## Materials and Methods

### Protein expression and purification.

The coding region corresponding to full-length WspR [24,48] was amplified by standard PCR using genomic DNA isolated from P. aeruginosa PAO1 (locus tag PA3702), P. syringae DC3000 (locus tag PSPTO_1499), P. fluorescence PfO-1 (locus tag Pfl_1058), or P. putida KT2440 (locus tag PP_1494), and was cloned into the pET21 expression plasmid (Novagen), yielding a C-terminally hexahistidine-tagged protein.

Transformed E. coli cells BL21(DE3) (Novagen) were grown in TB medium supplemented with 100 mg/l ampicillin at 37 °C. At a cell density corresponding to an absorbance of 1.0 at 600 nm, the temperature was reduced to 18 °C, and protein production was induced with 1 mM IPTG. Protein was expressed for 12–16 h. Cells were collected by centrifugation, resuspended in NiNTA buffer A (25 mM Tris-Cl [pH 8.2], 500 mM NaCl, 20 mM imidazole, and 5 mM 2-mercaptoethanol). After cell lysis by sonication, cell debris was removed by centrifugation at 40,000 × *g* for 1 h at 4 °C. Clear lysates were loaded onto HisTrap NiNTA columns (GE Healthcare) equilibrated in NiNTA buffer A. The resin was washed with 20 column volumes of NiNTA buffer A, and proteins were eluted on a gradient from 20 to 500 mM imidazole in NiNTA buffer A over 15 column volumes. Proteins were further subjected to SEC on a Superdex200 column (GE Healthcare) equilibrated in gel filtration buffer (25 mM Tris-Cl [pH 7.5], 100 mM NaCl, and 1 mM DTT). Fractions containing protein were pooled and concentrated on a Centricon ultrafiltration device (30 kDa cutoff; Millipore) to a final concentration of approximately 50 mg/ml. Protein aliquots were frozen in liquid nitrogen and stored at −80 °C.

Purified WspR with a C-terminal hexahistidine tag was analyzed by liquid chromatography/electrospray ionization mass spectrometry. The protein is free of detectable posttranslational modifications with a measured molecular weight (39,165.6 Da) in good agreement with the theoretical molar mass (39,165.4 Da).

Full-length SadR/RocR/PA3947 from P. aeruginosa PAO1 [49,50] was amplified as described above and cloned into a modified pProExHTb expression plasmid (Invitrogen), producing a Precision protease-cleavable N-terminally hexahistidine-tagged protein. Expression and purification were performed as described above with the addition of a Precision protease-cleavage step during purification for removal of the hexahistidine tag.

Nucleotide-free WspR^wt^ was prepared by treatment with the PDE SadR/RocR in the presence of 2 mM MgCl_2_ in a dialysis setup overnight at 4 °C. Progress was monitored by HPLC (see below). The PDE was removed by rebinding of WspR^wt^ to NiNTA matrix followed by elution and gel filtration as described above.

### Crystallization, X-ray data collection, and structure solution.

Crystals were obtained by hanging-drop vapor diffusion by mixing equal volumes of protein (5–30 mg/ml) and reservoir solution (0.1 M Tris-Cl [pH 8.0], 2.9 M NaCl, 15% xylitol) followed by incubation at 20 °C. Crystals appeared within 1 to 2 d, with typical dimensions of 0.2 mm × 0.2 mm × 0.2 mm. Crystals were flash-frozen in liquid nitrogen and kept at 100 K during data collection.

Crystallographic statistics for data collection are shown in Table S1. Datasets were collected using synchrotron radiation at the Cornell High Energy Synchrotron Source (CHESS, Ithaca, beamline A1). Data reduction was carried out with the software package HKL2000 [51]. The space group was determined to be C2 with *a* = 144.5 Å, *b* = 72.8 Å, *c* = 106.1.4 Å, and β = 110.8°. The asymmetric unit consists of two molecules, bound to four molecules of c-di-GMP and two magnesium ions. Phases were obtained from molecular replacement with the isolated GGDEF and CheY domains of PleD (PDB code: 1W25) [19] as search models using the software package Phenix [52]. The model of the WspR dimer was built manually starting from the molecular replacement solution. Refinement using CNS [53] and O [54] yielded the final model. Illustrations were made in Pymol (DeLano Scientific).

### Diguanylate cyclase and phosphodiesterase activity assays.

Two experimental systems for assaying diguanylate cyclase activity in vitro were used. A coupled spectrophotometric assay quantifies the amount of inorganic pyrophosphate, a product of the cyclization reaction, in solution (EnzChek Pyrophosphate Assay; Invitrogen) [55]. It relies on two enzymes, a pyrophosphatase that cleaves the pyrophosphate bond, producing two molecules of inorganic phosphate, and a purine nucleoside phosphorylase that converts 2-amino-6-mercapto-7-methylpurine ribonucleoside to ribose-1-phosphate and 2-amino-6-mercapto-7-methylpurine in a phosphate-dependent manner. Pyrophosphate production was monitored by a shift in the absorption peak from 330 nm to 360 nm. If not otherwise noted, WspR (0.5 μM) was incubated in the assay buffer containing 0.5 mM GTP and 2 mM MgCl_2_.

The second assay uses reverse-phase HPLC to separate nucleotides for the determination of nucleotide loading states of purified proteins and for the analysis of reaction products after incubation of proteins in the presence of 0.5 mM GTP and 2 mM MgCl_2_. (established by Abhishek Chatterjee, Cornell). Proteins were heat denatured at 95 °C for 5 min, followed by centrifugation for 10 min at 14,000 rpm. Supernatants were filtered through Microcon Centrifugal Filter units (Millipore) with a 10-kDa cutoff. Nucleotides were separated on a C18 reverse-phase column using a methanol-phosphate gradient (buffer A: 100 mM potassium phosphate [pH 6.0]; buffer B: 30% methanol/70% buffer A). Reaction products were collected and identified by comparison to standard nucleotides or by mass spectroscopy. Synthetic c-di-GMP was purchased from Biolog.

### Size-exclusion chromatography and SEC-coupled multiangle light scattering.

Purified protein (approximately 0.08 mM) was subjected to SEC using a Superdex 200 10/300 column (GE Healthcare) equilibrated in gel filtration buffer (25 mM Tris-Cl [pH 7.5], 100 mM NaCl, and 1 mM DTT).

For SEC-coupled multiangle light scattering [31], purified protein (approximately 0.1 mM) was subjected to SEC using a Shodex KW-803 column (JM Science) equilibrated in gel filtration buffer. The chromatography system was coupled to an 18-angle light-scattering detector (DAWN EOS) and refractive index detector (Optilab DSP) (Wyatt Technology). Data were collected every 0.5 s at a flow rate of 0.4 ml/min. Data analysis was carried out using the program ASTRA, yielding the molar mass and mass distribution (polydispersity) of the sample. For normalization of the light-scattering detectors and data quality control, monomeric bovine serum albumin (BSA; Sigma) was used.

### Cell-based diguanylate cyclase assay.

E. coli BL21(DE3) transformed with expression plasmids were grown in LB media supplemented with 100 mg/l ampicillin to a cell density corresponding to an absorbance of 0.5 at 600 nm. From each culture, 2.5 μl was spotted onto LB plates supplemented with 100 mg/l ampicillin, 50 mg/l Congo Red (CR; Sigma-Aldrich), and IPTG at the indicated concentrations. Plates were incubated at 30 °C overnight.

Purified proteins and cell lysates were analyzed by SEC on an analytical-scale Superdex200 10/300 column (GE Healthcare) equilibrated in gel filtration buffer. Fractions were analyzed by SDS-PAGE followed by Coomassie Blue staining or detection of the hexahistidine tag by western blotting.

## Supporting Information

Figure S1 Sequence Conservation of WspR in *Pseudomonas* and Related Species Sequence alignment of WspR. The sequence alignment of WspR proteins from various *Pseudomonas* and related species was generated using ClustalW [56] and formatted with ESPript using the Web server. Residues discussed in the text are highlighted with asterisks and arrows. The position of the conserved GGEEF motif (residues 251–255 in WspR from P. aeruginosa) is underlined (yellow box). Residues 140 to 171 have been predicted to form coiled-coil-like structures by multiple algorithms (http://www.expasy.org) (orange box). The following sequences were used to generate the alignment: P. aeruginosa PAO1, *P. entomophila* L48, P. fluorescens Sbw25, P. fluorescens PfO-1, P. fluorescens Pf-5, P. putida W619, P. syringae pv. syringae B728a, P. syringae pv. phaseolicola 1448A, P. syringae pv. tomato str. DC3000, Ralstonia metallidurans CH34, *R.eutropha* H16, Burkholderia phymatum STM815, B. dolosa AUO158, B. phytofirmans PsJN, Mesorhizobium loti MAFF303099, Burkholderia sp. 383, *B. ambifaria* MC40–6, and B. vietnamiensis G4.
(2.9 MB TIF)

Figure S2 Electron Density Maps of c-di-GMP-Bound WspR^wt^
(A) Electron density for the helical linker regions that connect the CheY and GGDEF domains of WspR at 2.40 Å. The electron density map shown has amplitudes of (|Fo| − |Fc|), with Fo and Fc being the observed and calculated structure factors. Phases were obtained by molecular replacement. The electron density contour is at 2.4 σ.
(B) Electron density for c-di-GMP at the I-site of the GGDEF domain. The electron density map shown has amplitudes of (|Fo| − |Fc|), with Fo and Fc being the observed and calculated structure factors. The electron density contour is at 3 σ.
(1.6 MB TIF)

Figure S3 Comparison of WspR with Structures of PleD and the Phospho-Receiver Domain Dimer of PhoB (A) Comparison of WspR with the structure of CheY domains of PhoB from E. coli. The structures of the CheY-homology domain dimer of WspR (residues 1–140) and a CheY domain dimer of PhoB (PDB code 1ZES) [26] were structurally aligned through superpositioning the CheY domain dimers.
(B) Comparison of dimeric WspR with the structure of PleD from C. crescentus. The structures of a WspR dimer (residues 1–140) and monomeric PleD (PDB code 1W25) [19] were aligned through superpositioning the CheY-homology domain dimer of WspR onto the intramolecular CheY-homology domain dimer of PleD.
(C) Comparison of tetrameric WspR with the monomeric and dimeric, activated structures of PleD from C. crescentus. The structures of monomeric PleD (left; PDB code 1W25) [19], and dimeric PleD (middle; PDB code 2V0N) [22] and WspR (right) are shown.
(2.6 MB TIF)

Figure S4 Structures Depicting Proposed Models for the Active, Intermediate, and Product-Inhibited State of WspR Driven by Stalk Interactions (A) Proposed structure closely resembling an active state of WspR. The two molecules in the asymmetric unit form a dimer via pairing of their CheY-like phospho-receiver domains reminiscent of an active CheY dimer [26]. In the crystal, the tips of the stalks are splayed apart by a symmetry-related dimer (see [B]). Sequence-based analysis predicts a bona fide coiled-coil structure for residues 140–171 (Figure S1), suggesting that the stalks might convene in the active conformation bringing the GGDEF domains into close proximity. The boxed areas correspond to the close-up views shown in (D). The maximal dimensions for the various complexes are shown.
(B) Tetrameric assembly consisting of two symmetry-related dimers. Two C2-symmetry–related crystallographic dimers of WspR are shown intertwined in a head-to-head orientation. The stalks form a tetrameric structure splaying apart the coiled-coils and physically blocking the active sites. The GGDEF domains are linked via c-di-GMP molecules that bridge the I-sites of neighboring molecules. Arrows indicate how breaking up the CheY domain dimers accompanied by a rigid body rotation of the CheY-stalk module would facilitate the formation of two identical dimers shown in (C) (elongated dimer 1). Such a motion is similar to the one shown in Figure 1C.
(C) Possible elongated dimer states derived from the tetrameric assembly. Cyclic di-GMP-bound, inactive WspR is dimeric in solution but crystallizes as a tetrameric assembly. From the three possible elongated WspR dimers that can be derived from the tetrameric structure (Figure S4B), the elongated dimer 1 (also shown in Figure 8A, right panel) might represent a structure close to the conformation of inactive WspR in solution, based on structural and functional arguments presented in the main text. Briefly, two symmetry-related molecules shown in color (chain A) of WspR in the elongated dimer 1 are held together by three interfaces: stalk-stalk, stalk-GGDEF domain, and GGDEF domain-CheY domain of adjacent molecules. In the stalks, the same residues that would satisfy the coiled-coil and drive tetramerization form the hydrophobic center of the dimer (D–F). The ionic interaction between the GGEEF motif and the stalk is shown in detail (close-up view in inset). Elongated dimer 2 is mediated exclusively by antiparallel packing of the stalks, burying the smallest surface area in the dimer interface compared to dimers 1 and 3. Other interactions observed in elongated dimer 1 have not formed yet in elongated dimer 2. Considering the bridging of the GGDEF domains by c-di-GMP, elongated dimer 3 might be an intuitive candidate for the inhibited state (Figure S4C, right panel). However, the dissociation of such a dimer from the tetramer would be sterically hindered. In addition, the buried surface area at the interface (1,518 Å^2^ surface are buried between the two chains, 830 Å^2^ of which are buried between the GGDEF domains) is less extensive compared to elongated dimer 1 (2,430 Å^2^), and the interaction between the GGDEF domains appears to be functionally less important since a mutation at the interface (WspR^R195E^) has no effect on the protein, being mechanistically indistinguishable from wild-type WspR (unpublished data). These arguments and considerations support the hypothesis that elongated dimer 1 might represent a conformation close to the inhibited state in solution that assembled to the tetrameric assembly in the crystal lattice. Buried surface areas were calculated in CNS [53].
(D) Close-up view of the tip of the stalks considering a compact dimer state. Asterisks indicate residues targeted for site-directed mutagenesis (see Figure S6). The arrow indicates how the coiled-coil might convene in the active state.
(E) Close-up view of the tip of the stalks in the tetrameric state. The area boxed in (B) is shown as a close-up view. Leucine 170 (green sphere) and leucine 167 (pink sphere) are colored as in (D).
(F) Close-up view of the tip of the stalks in an antiparallel, elongated dimer 1. The area boxed in (C) is shown as a close-up view.
(2.3 MB TIF)

Figure S5 Conservation of the Oligomeric Switching Mechanism in WspR from Other *Pseudomonas* Species (A) Sequence conservation mapped onto the surface of WspR. The structure of a WspR dimer is shown. The surface of molecule A is shown and presented as a color gradient from green to grey to red (0%–100% sequence conservation). Identical residues are colored in red. Molecule B is shown as grey Cα trace.
(B) ClustalW scores for pairwise comparisons of WspR sequences from P. aeruginosa, *putida*, *fluorescence*, and *syringae*. Protein sequence identities of WspR species analyzed in (C–E) were calculated using ClustalW [56].
(C) Detection of guanosine nucleotides bound to WspR by a reverse-phase HPLC-based assay. Chromatograms for pure c-di-GMP (standard) are shown as dashed, light-grey line. WspR^wt^ from the indicated *Pseudomonas* species expressed in E. coli purifies with c-di-GMP bound (red trace). Nucleotide-free WspR^wt^ was obtained by including PDE treatment in the purification protocol followed by removal of pGpG (green trace).
(D) PDE treatment triggers a conformational change in WspR. Proteins were analyzed by SEC. Cyclic di-GMP–bound WspR^wt^ (0.24 mM) (red trace) was incubated with PDE (0.008 mM) in gel filtration buffer supplemented with 10 mM Mn^2+^ for 2 h at 25 °C (dark-grey trace). Nucleotide-free WspR^wt^ was further purified by removal of PDE and pGpG, followed by preparative gel filtration and concentration (green trace).
(E) Comparison of enzymatic activity of c-di-GMP-bound and nucleotide-free WspR. Nucleotide-bound (red bars) or nucleotide-free (green bars) WspR^wt^ (0.5 μM) was incubated at 25 °C in assay buffer (EnzChek Pyrophosphate Assay; Invitrogen) and pyrophosphate production was measured by continuously monitoring absorbance at 360 nm. Error bars indicate standard deviations of three independent experiments.
(1.9 MB TIF)

Figure S6 Characterization of Stalk Mutants of WspR (A) Close-up view of the stalk region in the tetrameric assembly of WspR. In the crystal, two WspR dimers interact in a head-to-head fashion involving the tip of the stalk regions. Leucine residues targeted for mutagenesis (changed to aspartates; L170D and L167D) are central to the interactions driven by the stalks in the dimeric and tetrameric assemblies (see Figure S4).
(B) Stalk mutants of WspR purify c-di-GMP bound. Nucleotides bound to proteins with mutations at the tip of the stalks (WspR^L170D^ or WspR^L167D^; purple and blue trace, respectively) expressed in E. coli were analyzed using a reverse-phase HPLC assay.
(C) SEC profiles of mutant and wild-type WspR. Nucleotide-bound and nucleotide-free WspR^wt^ (red and green trace, respectively), WspR^L170D^ (purple trace), and WspR^L167D^ (blue trace) (0.24 mM) were analyzed by analytical gel filtration in gel filtration buffer. Maximum peak heights correspond to elution volumes of 11.7, 12.1, and 13.3 ml.
(D) Comparison of enzymatic activity of wild-type and mutant forms of WspR. WspR^wt^ (nucleotide-bound or -free) or mutant variants (WspR^L170D^ or WspR^L167D^) (0.5 μM) were incubated at 25 °C in assay buffer (EnzChek Pyrophosphate Assay; Invitrogen) and pyrophosphate production was measured by continuously monitoring absorbance at 360 nm. Coloring corresponds to the scheme shown in (C). Error bars indicate standard deviations of three independent experiments. Although WspR^L167D^ is predominantly monomeric and has no activity under the conditions used here, it purifies with c-di-GMP bound. Upon overexpression, protein levels are likely to be high enough to facilitate the formation of transient dimers exhibiting diguanylate cyclase activity, similar to the activity observed for the isolated GGDEF domain at high expression levels (Figure 6A and data not shown).
(1.3 MB TIF)

Table S1 Data Collection and Refinement Statistics (44 KB DOC)

### Accession Numbers

Atomic coordinates and structure factors for P. aeruginosa PA3702 have been deposited in the RCSB Protein Data Bank (http://www.rcsb.org/pdb) under ID code 3BRE.

The National Center for Biotechnology Information (NCBI) (http://www.ncbi.nlm.nih.gov) accession numbers for the protein sequences used for alignment of WspR are as follows: Burkholderia sp. 383 (YP_372775), B. ambifaria MC40–6 (ZP_01553618), B. dolosa AUO158 (ZP_00984936), B. phytofirmans PsJN (ZP_01511812), B. vietnamiensis G4 (YP_001117051)., B. phymatum STM815 (ZP_01501464), Mesorhizobium loti MAFF303099 (NP_109384), P. aeruginosa PAO1 (NP_252391), P. entomophila L48 (YP_606955), P. fluorescens Pf-5 (YP_258266), P. fluorescens PfO-1 (YP_346790), P. fluorescens Sbw25 (AAL71852), P. putida W619 (ZP_01639796), P. syringae pv. phaseolicola 1448A (YP_276013), P. syringae pv. syringae B728a (YP_234398), P. syringae pv. tomato str. DC3000 (NP_791325), Ralstonia eutropha H16 (YP_841562), and R. metallidurans CH34 (YP_586104).

## Abbreviations

c-di-GMP: cyclic dimeric guanosine monophosphate
GTP: guanosine triphosphate
HPLC: high-performance liquid chromatography
I-site: inhibitory site
PDE: phosphodiesterase
SEC: size-exclusion chromatography
