# Supplementary figures and images for: Phosphorylation-Independent Regulation of the Diguanylate Cyclase WspR
Source: PLoS Biol. 2008 Mar 25;6(3):e67. doi: 10.1371/journal.pbio.0060067 (PMC2270323; doi:10.1371/journal.pbio.0060067)

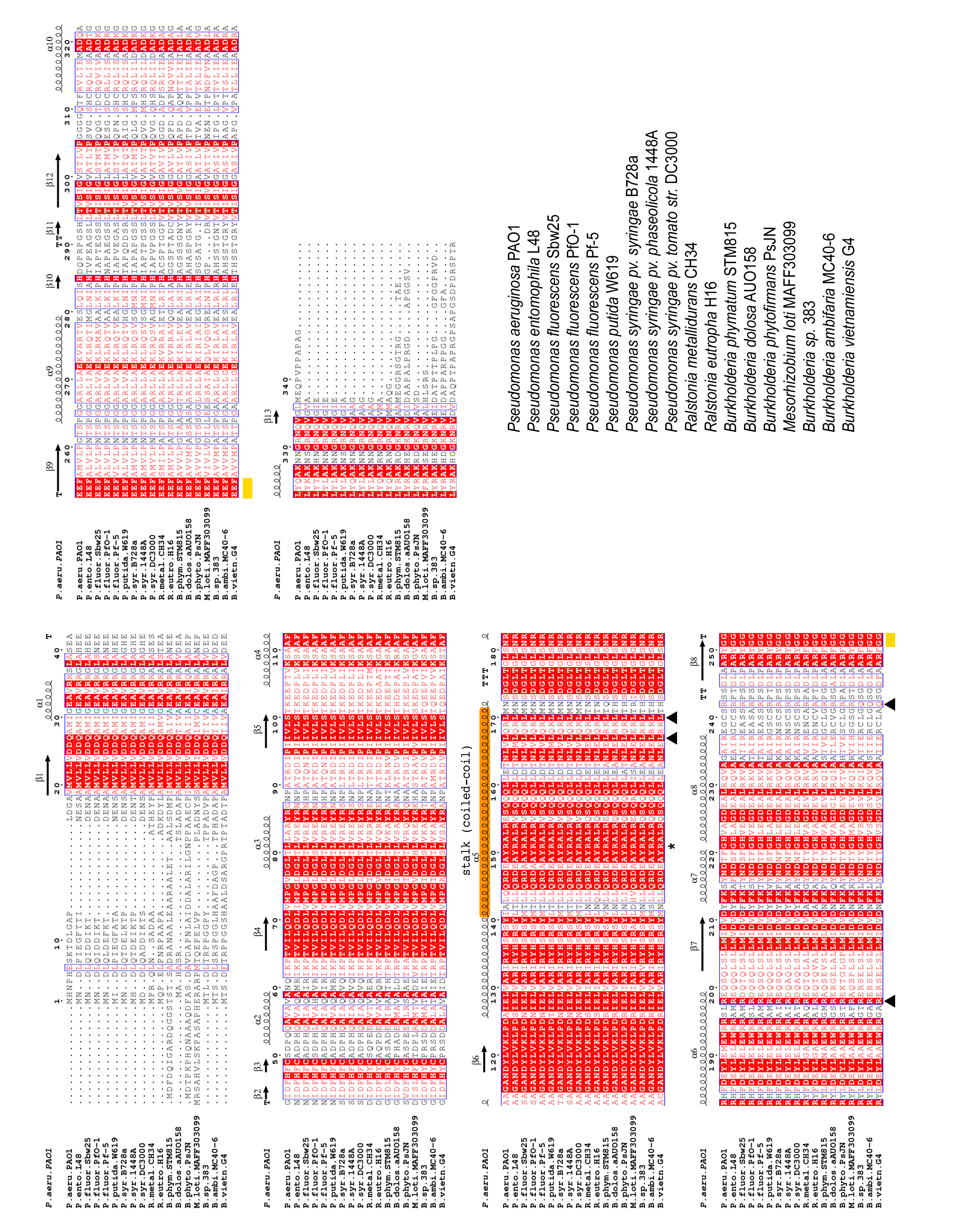

Supplement: Figure S1 — Sequence alignment of WspR. The sequence alignment of WspR proteins from various Pseudomonas and related species was generated using ClustalW [56] and formatted with ESPript using the Web server. Residues discussed in the text are highlighted with asterisks and arrows. The position of the conserved GGEEF motif (residues 251–255 in WspR from P. aeruginosa) is underlined (yellow box). Residues 140 to 171 have been predicted to form coiled-coil-like structures by multiple algorithms (http://www.expasy.org) (orange box). The following sequences were used to generate the alignment: P. aeruginosa PAO1, P. entomophila L48, P. fluorescens Sbw25, P. fluorescens PfO-1, P. fluorescens Pf-5, P. putida W619, P. syringae pv. syringae B728a, P. syringae pv. phaseolicola 1448A, P. syringae pv. tomato str. DC3000, Ralstonia metallidurans CH34, R.eutropha H16, Burkholderia phymatum STM815, B. dolosa AUO158, B. phytofirmans PsJN, Mesorhizobium loti MAFF303099, Burkholderia sp. 383, B. ambifaria MC40–6, and B. vietnamiensis G4. (2.9 MB TIF) [file pbio.0060067.sg001.tif]

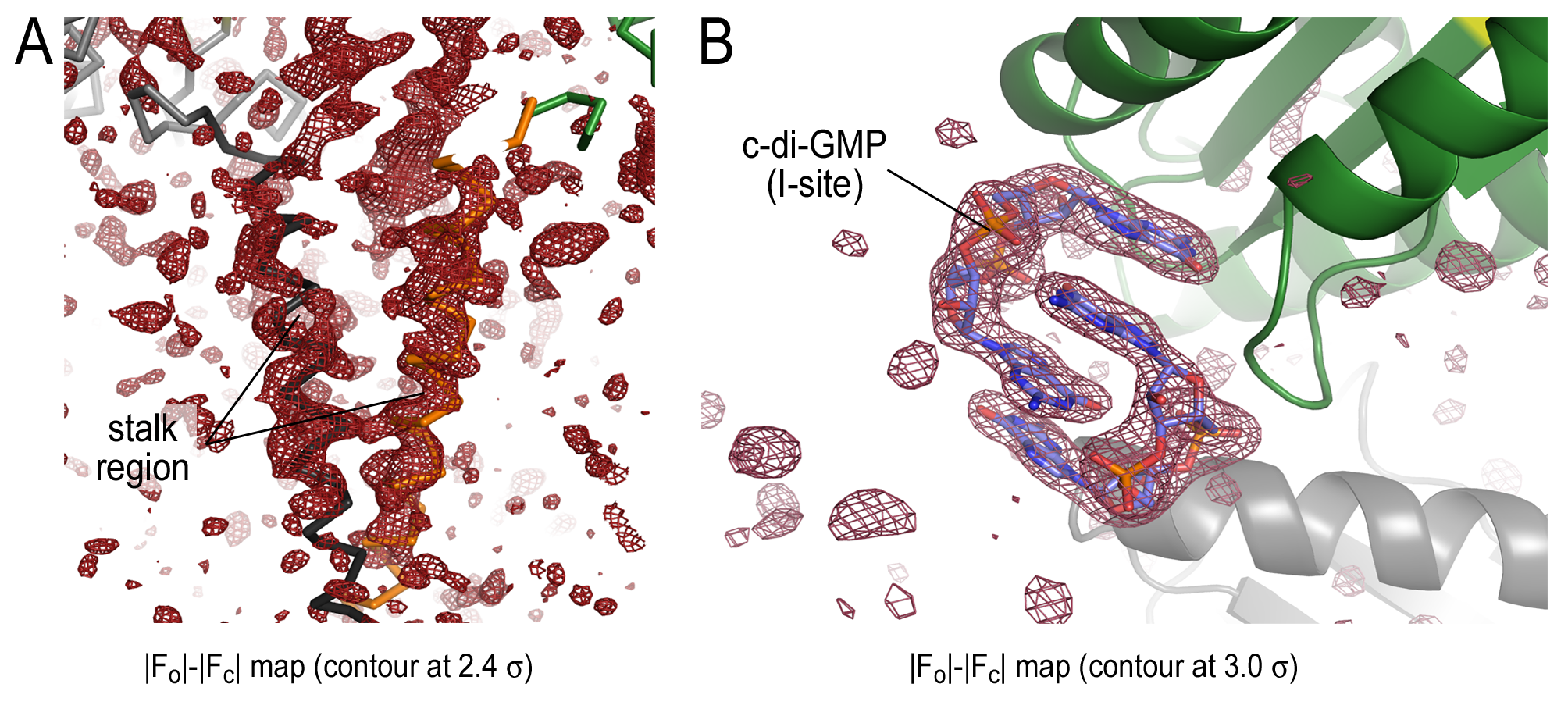

Supplement: Figure S2 — (A) Electron density for the helical linker regions that connect the CheY and GGDEF domains of WspR at 2.40 Å. The electron density map shown has amplitudes of (|Fo| − |Fc|), with Fo and Fc being the observed and calculated structure factors. Phases were obtained by molecular replacement. The electron density contour is at 2.4 σ. (B) Electron density for c-di-GMP at the I-site of the GGDEF domain. The electron density map shown has amplitudes of (|Fo| − |Fc|), with Fo and Fc being the observed and calculated structure factors. The electron density contour is at 3 σ. (1.6 MB TIF) [file pbio.0060067.sg002.tif]

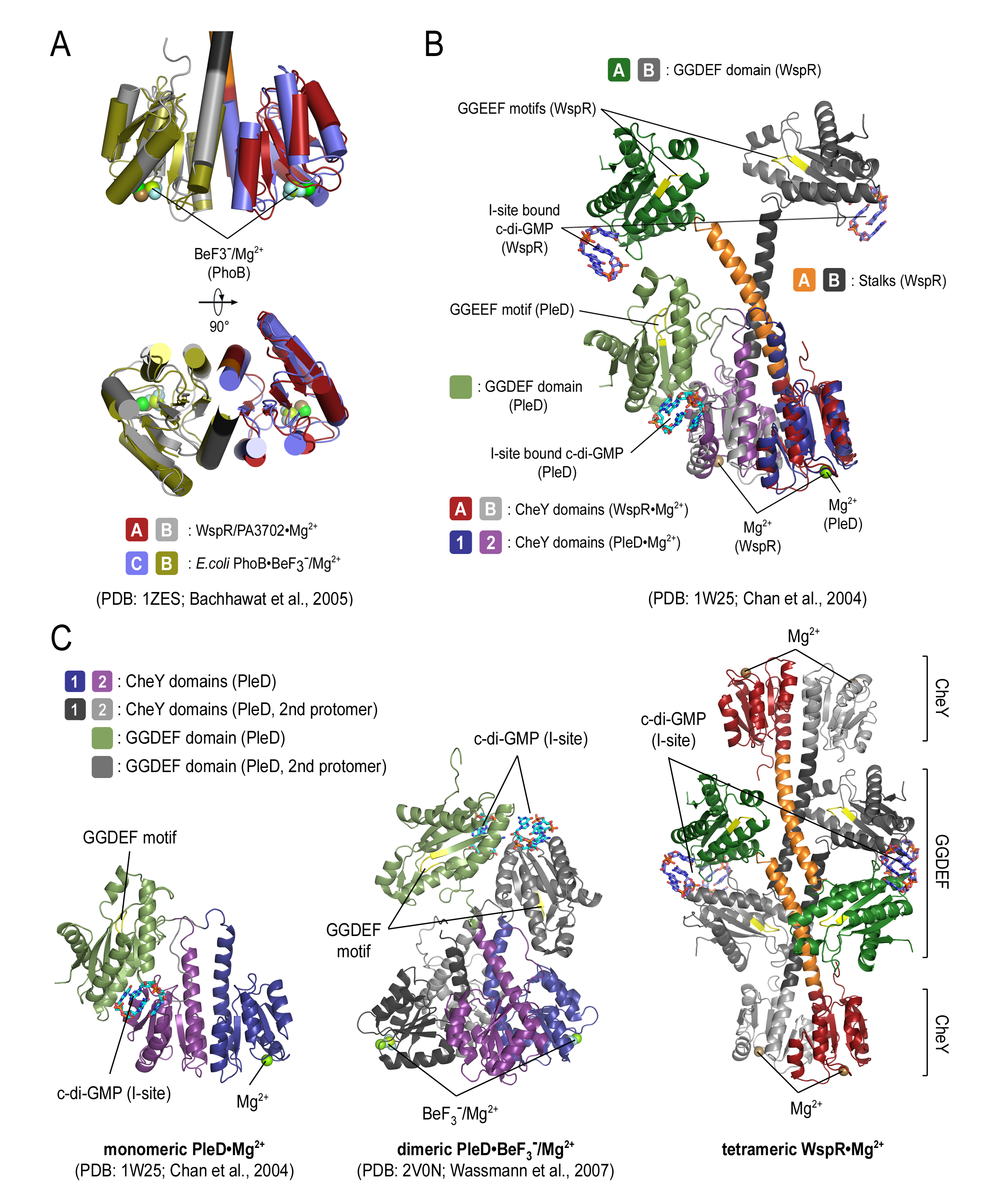

Supplement: Figure S3 — (A) Comparison of WspR with the structure of CheY domains of PhoB from E. coli. The structures of the CheY-homology domain dimer of WspR (residues 1–140) and a CheY domain dimer of PhoB (PDB code 1ZES) [26] were structurally aligned through superpositioning the CheY domain dimers. (B) Comparison of dimeric WspR with the structure of PleD from C. crescentus. The structures of a WspR dimer (residues 1–140) and monomeric PleD (PDB code 1W25) [19] were aligned through superpositioning the CheY-homology domain dimer of WspR onto the intramolecular CheY-homology domain dimer of PleD. (C) Comparison of tetrameric WspR with the monomeric and dimeric, activated structures of PleD from C. crescentus. The structures of monomeric PleD (left; PDB code 1W25) [19], and dimeric PleD (middle; PDB code 2V0N) [22] and WspR (right) are shown. (2.6 MB TIF) [file pbio.0060067.sg003.tif]

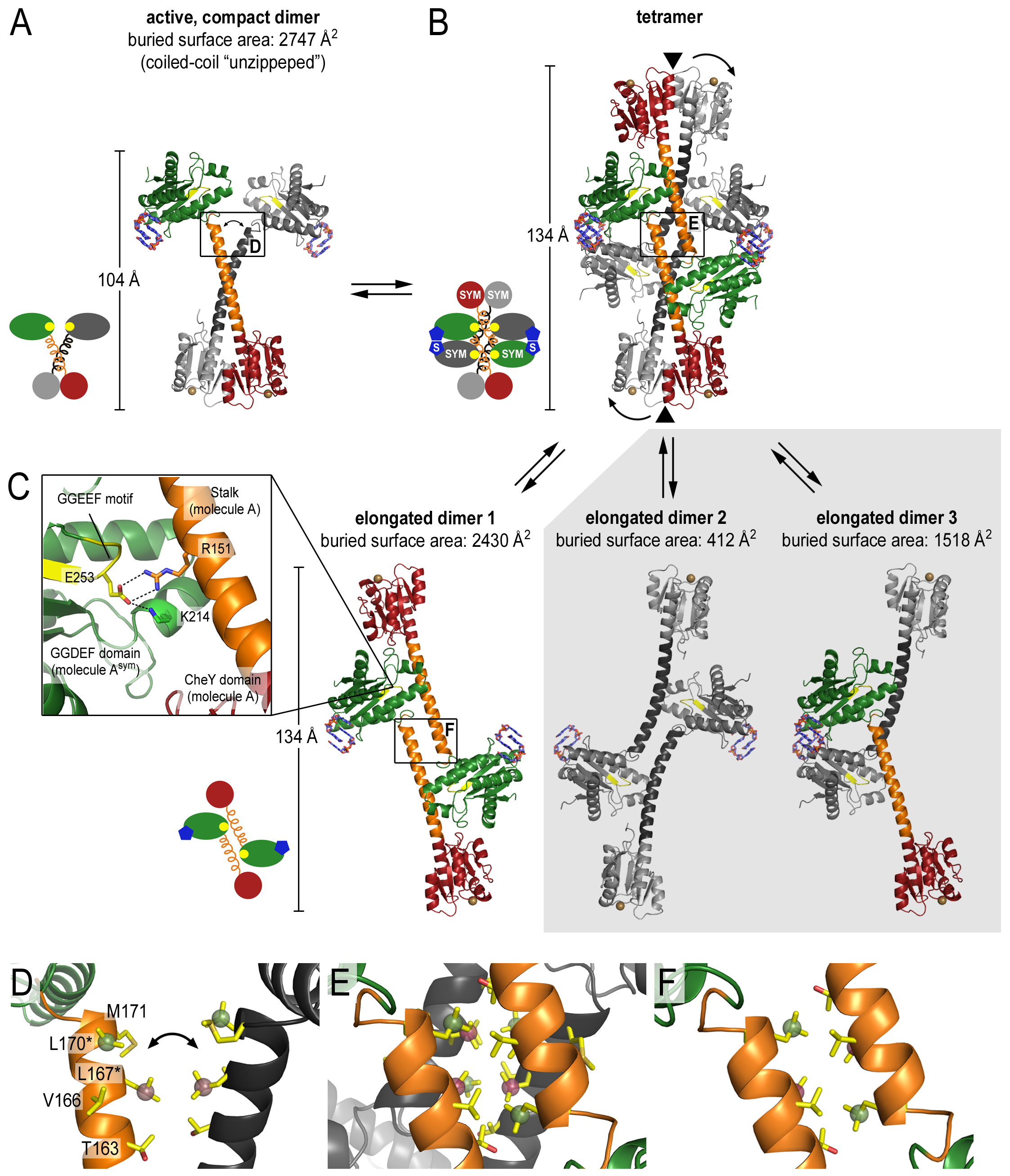

Supplement: Figure S4 — (A) Proposed structure closely resembling an active state of WspR. The two molecules in the asymmetric unit form a dimer via pairing of their CheY-like phospho-receiver domains reminiscent of an active CheY dimer [26]. In the crystal, the tips of the stalks are splayed apart by a symmetry-related dimer (see [B]). Sequence-based analysis predicts a bona fide coiled-coil structure for residues 140–171 (Figure S1), suggesting that the stalks might convene in the active conformation bringing the GGDEF domains into close proximity. The boxed areas correspond to the close-up views shown in (D). The maximal dimensions for the various complexes are shown. (B) Tetrameric assembly consisting of two symmetry-related dimers. Two C2-symmetry–related crystallographic dimers of WspR are shown intertwined in a head-to-head orientation. The stalks form a tetrameric structure splaying apart the coiled-coils and physically blocking the active sites. The GGDEF domains are linked via c-di-GMP molecules that bridge the I-sites of neighboring molecules. Arrows indicate how breaking up the CheY domain dimers accompanied by a rigid body rotation of the CheY-stalk module would facilitate the formation of two identical dimers shown in (C) (elongated dimer 1). Such a motion is similar to the one shown in Figure 1C. (C) Possible elongated dimer states derived from the tetrameric assembly. Cyclic di-GMP-bound, inactive WspR is dimeric in solution but crystallizes as a tetrameric assembly. From the three possible elongated WspR dimers that can be derived from the tetrameric structure (Figure S4B), the elongated dimer 1 (also shown in Figure 8A, right panel) might represent a structure close to the conformation of inactive WspR in solution, based on structural and functional arguments presented in the main text. Briefly, two symmetry-related molecules shown in color (chain A) of WspR in the elongated dimer 1 are held together by three interfaces: stalk-stalk, stalk-GGDEF domain, and GGDEF domain-C [file pbio.0060067.sg004.tif]

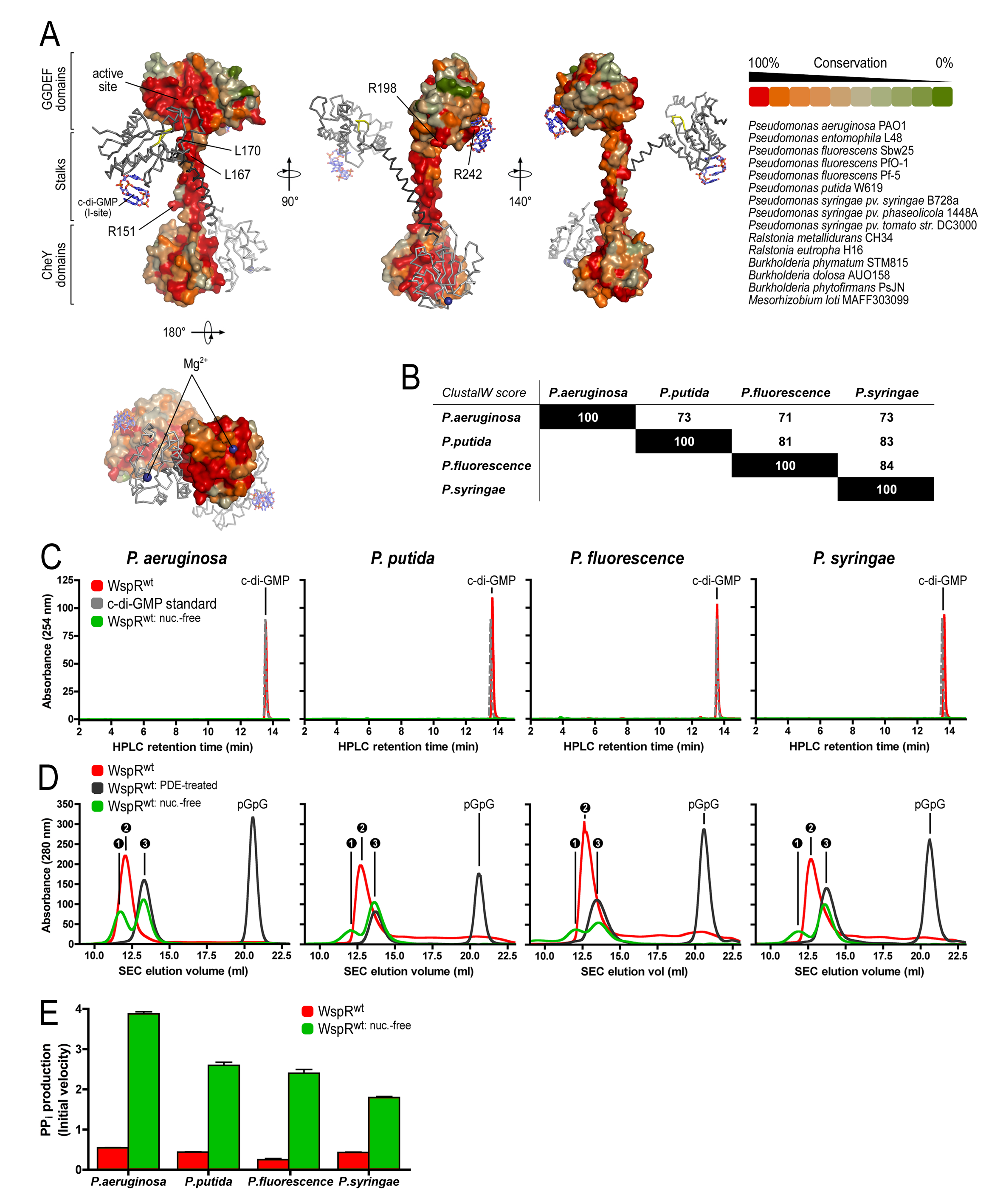

Supplement: Figure S5 — (A) Sequence conservation mapped onto the surface of WspR. The structure of a WspR dimer is shown. The surface of molecule A is shown and presented as a color gradient from green to grey to red (0%–100% sequence conservation). Identical residues are colored in red. Molecule B is shown as grey Cα trace. (B) ClustalW scores for pairwise comparisons of WspR sequences from P. aeruginosa, putida, fluorescence, and syringae. Protein sequence identities of WspR species analyzed in (C–E) were calculated using ClustalW [56]. (C) Detection of guanosine nucleotides bound to WspR by a reverse-phase HPLC-based assay. Chromatograms for pure c-di-GMP (standard) are shown as dashed, light-grey line. WspRwt from the indicated Pseudomonas species expressed in E. coli purifies with c-di-GMP bound (red trace). Nucleotide-free WspRwt was obtained by including PDE treatment in the purification protocol followed by removal of pGpG (green trace). (D) PDE treatment triggers a conformational change in WspR. Proteins were analyzed by SEC. Cyclic di-GMP–bound WspRwt (0.24 mM) (red trace) was incubated with PDE (0.008 mM) in gel filtration buffer supplemented with 10 mM Mn2+ for 2 h at 25 °C (dark-grey trace). Nucleotide-free WspRwt was further purified by removal of PDE and pGpG, followed by preparative gel filtration and concentration (green trace). (E) Comparison of enzymatic activity of c-di-GMP-bound and nucleotide-free WspR. Nucleotide-bound (red bars) or nucleotide-free (green bars) WspRwt (0.5 μM) was incubated at 25 °C in assay buffer (EnzChek Pyrophosphate Assay; Invitrogen) and pyrophosphate production was measured by continuously monitoring absorbance at 360 nm. Error bars indicate standard deviations of three independent experiments. (1.9 MB TIF) [file pbio.0060067.sg005.tif]

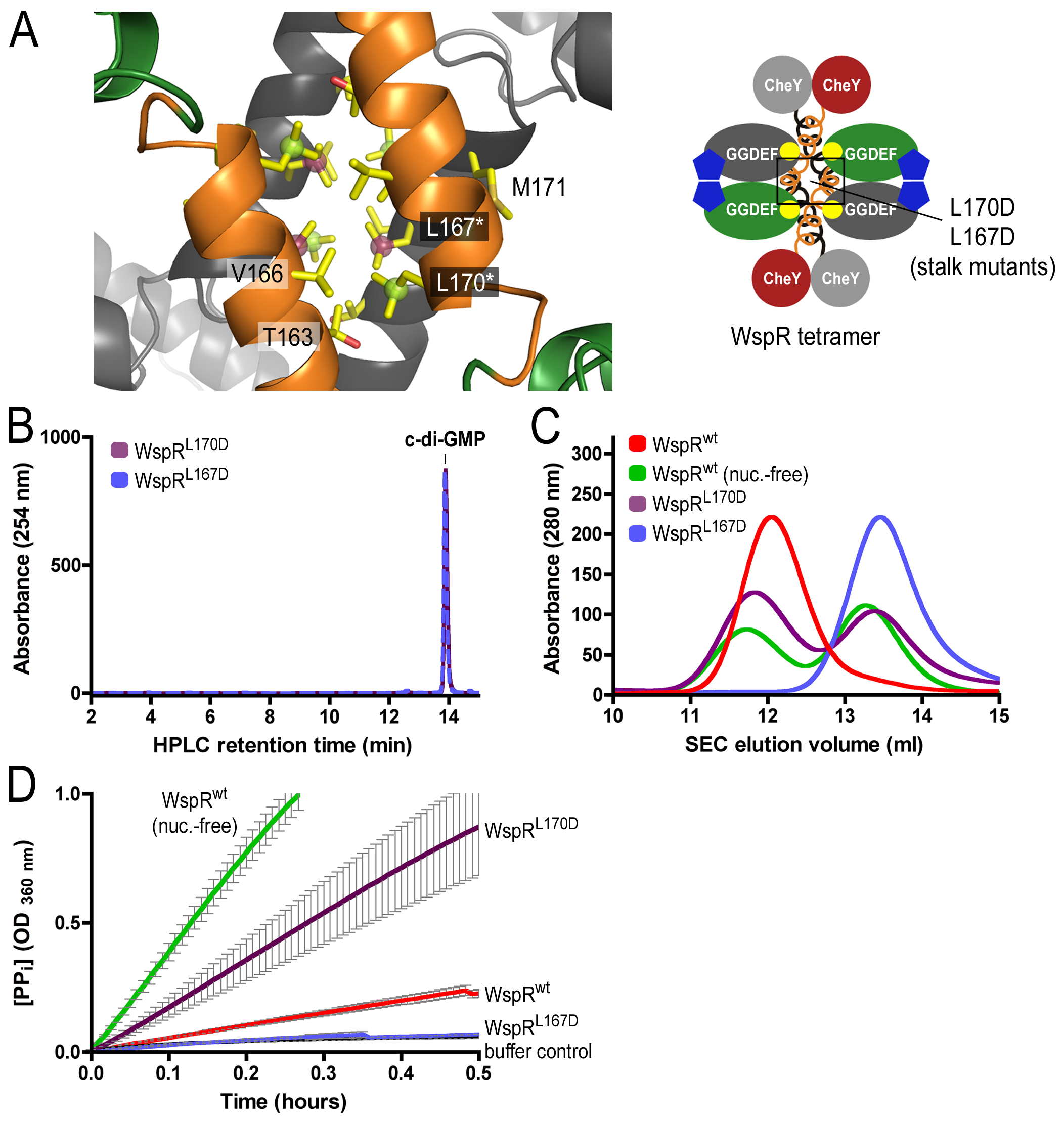

Supplement: Figure S6 — (A) Close-up view of the stalk region in the tetrameric assembly of WspR. In the crystal, two WspR dimers interact in a head-to-head fashion involving the tip of the stalk regions. Leucine residues targeted for mutagenesis (changed to aspartates; L170D and L167D) are central to the interactions driven by the stalks in the dimeric and tetrameric assemblies (see Figure S4). (B) Stalk mutants of WspR purify c-di-GMP bound. Nucleotides bound to proteins with mutations at the tip of the stalks (WspRL170D or WspRL167D; purple and blue trace, respectively) expressed in E. coli were analyzed using a reverse-phase HPLC assay. (C) SEC profiles of mutant and wild-type WspR. Nucleotide-bound and nucleotide-free WspRwt (red and green trace, respectively), WspRL170D (purple trace), and WspRL167D (blue trace) (0.24 mM) were analyzed by analytical gel filtration in gel filtration buffer. Maximum peak heights correspond to elution volumes of 11.7, 12.1, and 13.3 ml. (D) Comparison of enzymatic activity of wild-type and mutant forms of WspR. WspRwt (nucleotide-bound or -free) or mutant variants (WspRL170D or WspRL167D) (0.5 μM) were incubated at 25 °C in assay buffer (EnzChek Pyrophosphate Assay; Invitrogen) and pyrophosphate production was measured by continuously monitoring absorbance at 360 nm. Coloring corresponds to the scheme shown in (C). Error bars indicate standard deviations of three independent experiments. Although WspRL167D is predominantly monomeric and has no activity under the conditions used here, it purifies with c-di-GMP bound. Upon overexpression, protein levels are likely to be high enough to facilitate the formation of transient dimers exhibiting diguanylate cyclase activity, similar to the activity observed for the isolated GGDEF domain at high expression levels (Figure 6A and data not shown). (1.3 MB TIF) [file pbio.0060067.sg006.tif]
