# Supplementary material for: Phosphorylation-Independent Regulation of the Diguanylate Cyclase WspR
Source: PLoS Biol. 2008 Mar 25;6(3):e67. doi: 10.1371/journal.pbio.0060067 (PMC2270323; doi:10.1371/journal.pbio.0060067)
Supplement: Table S1 — (44 KB DOC) [file pbio.0060067.st001.doc]

| Supplemental Table 1. Data Collection and Refinement Statistics | |
| --- | --- |
| **Structure** | WspR full-length |
|  | **native** |
| Space Group | C2 |
| Unit Cell | a=144.5 Å, b=72.8 Å, c=106.1 Å |
|  | α=γ=90°, β=110.8° |
| X-ray source | CHESS (A1) |
| Wavelength (Å) | 0.977 |
| Resolution (Å) | 50-2.39 (2.48-2.39) |
| Measured reflections (#) | 171,005 |
| Unique reflect. (#) | 40,110 |
| Data redundancy | 4.3 (3.0) |
| Completeness (%) | 98.9 (94.5) |
| R_sym_ (%) | 6.6 (45.9) |
| I / σ_I_ | 16.2 (2.0) |
| **Current Model Refinement Statistics^a^** | |
| Phasing | Molecular Replacement |
| Molecules/AU | 2 (no NCS applied)^c^ |
| R_work_ / R_free_ (%)^b^ | 24.2 / 27.8 |
| Free R test set size (#/%) | 2707/6.7 |
| Number of protein atoms | 4996 |
| Number of hetero-atoms | 186 |
| Rmsd bond length (Å) | 0.008 |
| Rmsd bond angles (°) | 1.4 |
| Rmsd B factors (Å^2^) (main chain/side chain) | 1.44/2.04 |
| ^a^Values as defined in SCALEPACK [51] and CNS [53]; ^b^No sigma cutoffs; ^c^: Non-crystallographic symmetry was not applied. | |
